# Genetic Susceptibility Toward Nausea and Vomiting in Surgical Patients

**DOI:** 10.3389/fgene.2021.816908

**Published:** 2022-01-31

**Authors:** Yvonne Gloor, Christoph Czarnetzki, François Curtin, Béatrice Gil-Wey, Martin R. Tramèr, Jules A. Desmeules

**Affiliations:** ^1^ Division of Clinical Pharmacology and Toxicology, Department of Anesthesiology, Pharmacology, Intensive Care and Emergency Medicine, Geneva University Hospitals (HUG), Geneva, Switzerland; ^2^ Division of Anesthesiology, Department of Anesthesiology, Pharmacology, Intensive Care and Emergency Medicine, Geneva University Hospitals (HUG), Geneva, Switzerland; ^3^ Division of Anesthesiology, Department of Anesthesiology, Intensive Care and Emergency Medicine, Ospedale Regionale di Lugano, Ente Ospedaliero Cantonale, Lugano, Switzerland; ^4^ Personalized Health Programs, Swiss Federal Institute of Technology Zurich (ETHZ), Zurich, Switzerland

**Keywords:** PONV, genetic risk factors, *HTR3B* polymorphisms, risk score, serotonin receptor

## Abstract

Postoperative nausea and vomiting (PONV) are frequently occurring adverse effects following surgical procedures. Despite predictive risk scores and a pallet of prophylactic antiemetic treatments, it is still estimated to affect around 30% of the patients, reducing their well-being and increasing the burden of post-operative care. The aim of the current study was to characterize selected genetic risk factors of PONV to improve the identification of at risk patients. We genotyped 601 patients followed during the first 24 h after surgery for PONV symptoms in the absence of any antiemetic prophylaxis. These patients were recruited in the frame of a randomized, placebo controlled clinical study aiming to test the efficacy of dexamethasone as a treatment of established PONV. We examined the impact of selected single nucleotide polymorphisms (SNPs) located around 13 different genes and the predicted activity of 6 liver drug metabolizing enzymes from the cytochromes P450 family (CYP) on the occurrence and recurrence of PONV. Our genetic study confirms the importance of genetic variations in the type 3B serotonin receptor in the occurrence of PONV. Our modelling shows that integration of *rs3782025* genotype in preoperative risk assessments may help improve the targeting of antiemetic prophylaxis towards patients at risk of PONV.

## Introduction

Postoperative nausea and vomiting (PONV) are frequently occurring adverse effects following surgical procedures concerning about one third of patients, with a prevalence that can reach up to 70–80% in high risk populations (Amirshahi et al., 2020; Elvir-Lazo et al., 2020; Ziemann-Gimmel et al., 2020). PONV considerably affects patient well-being, increases recovery time and cost through enhanced care and potential secondary effects. The most effective and commonly used anti-emetic treatments consist of 5-HT_3_ and D_2_-and more recently NK-1 receptor antagonists [reviewed in (Elvir-Lazo et al., 2020; Gan et al., 2020)]. Corticosteroids, especially dexamethasone, complete the spectrum of clinically potent preventive anti-emetic medications (Chu et al., 2014). As systematic administration of antiemetic prophylaxis before surgery is neither safe nor cost-effective (Tramèr et al., 1999), it is important to identify patients with a high-risk profile. A number of patient characteristics, as well as anesthetic or surgical procedures have been identified as risk factors of PONV including, but not limited to: female gender, young age, non-smoking status, previous history of PONV, use of volatile anesthetics, visceral and gynecological procedures, and perioperative opioid consumption (Palazzo and Evans, 1993; Heyland et al., 1997; Koivuranta et al., 1997; Apfel et al., 1999; Sinclair et al., 1999; Junger et al., 2001; Murphy et al., 2006; Leslie et al., 2008).

The contribution of individual genetic susceptibility to PONV was first supported by the notion of family history of PONV, found to be a significant risk factor in pediatric patients (Eberhart et al., 2004) as well as a potential role of ethnicity that remains however controversial [reviewed in (Gan, 2006)]. Identification of genetic determinants playing a role in nausea and vomiting has been attempted using both candidate gene approaches and genome-wide association (GWAS) techniques in the context of postoperative as well as chemotherapy-induced (CINV) or pregnancy-related (NVP) nausea and vomiting. Candidate genes were mainly selected from signaling pathways known to be involved in emesis such as serotonin (5-HT), dopamine, acetylcholine, and neurokinin-1 (substance P) [reviewed in (Hornby, 2001; Horn et al., 2014; Janicki and Sugino, 2014)]. Research focusing on opioid-induced nausea and vomiting (OINV), uncovered additional polymorphism of interest as opioids are often used to manage perioperative analgesia and belong themselves to the recognized risk factors of PONV (Smith et al., 2012).

The pro-emetic role of serotonin and the efficiency of antagonists of the type 3 serotonin receptors (5-HTR_3_), such as ondansetron, as anti-emetic treatment, has prompted a number of studies on the effect of polymorphisms affecting serotonin receptor genes on nausea and vomiting. Several studies uncovered significant associations between *HTR3A* and *HTR3B* related genetic polymorphisms and PONV (Rueffert et al., 2009; Laugsand et al., 2011; Lehmann et al., 2013; Ma et al., 2013; Yan et al., 2021). Genetic variations affecting other serotonin receptors such as *5-HTR*_*2A*_, might also participate in PONV susceptibility (Kato et al., 2006). Other mutations significantly associated with the occurrence of nausea and vomiting have been found in the type 2 dopamine receptor (*DRD2*) (Nakagawa et al., 2008; Frey et al., 2016), the neurokinin-1 receptor (*TACR1*) (Hayase et al., 2015), the type 3 muscarinic acetylcholine receptor (*CHRM3*) (Janicki et al., 2011; Laugsand et al., 2011; Klenke et al., 2018) or the cathechol O-methyltransferase (*COMT*) gene responsible for degradation of catecholamines (Kolesnikov et al., 2011; Laugsand et al., 2011; Wesmiller et al., 2017).

Besides differences in pro- and anti-emetic signal transduction, variations in the mu type opioid receptor (*OPRM1*) (Kolesnikov et al., 2011; Kong et al., 2018; Aroke and Hicks, 2019) and the efflux transporter encoded by *ABCB1* acting at the blood brain barrier on a broad range of substrate (Coulbault et al., 2006; Zwisler et al., 2010) could contribute to individual susceptibility toward PONV, either directly by their modulation of endogenous signals or through their role in the processing of exogenous opioids and, for *ABCB1*, surgeries or anesthetic agents. Polymorphisms in the fatty acid amide hydrolase (*FAAH*) which degrades endocannabinoids responsible for the mediation of the anti-nociceptive effects of morphine have, also been associated with PONV (Sadhasivam et al., 2015).

In addition, drug metabolizing enzyme (DME) might also participate to patient-related PONV risk factors. This contribution was first suggested by Sweeney (Tornio and Backman, 2018) in the context of the recognized protective effect of smoking on PONV (Sweeney, 2002). Indeed, although a direct link between metabolic activity and PONV has not yet been demonstrated, DMEs and in particular cytochromes from the P450 family (CYP) show a large inter-individual variability in activity resulting both from genetic and environmental origin including smoking [reviewed in (Zanger and Schwab, 2013; Tornio and Backman, 2018)]. The role of CYP activity variability in the context of PONV has most commonly focused on differences in anti-emetic drug metabolism (Candiotti et al., 2005; Nielsen and Olsen, 2008; Wesmiller et al., 2013; Aroke and Hicks, 2019). One example is the metabolism of the 5-HTR_3_ receptor antagonist ondansetron by CYP1A2 and CYP3A (Huddart et al., 2019). However, in addition, to a direct detoxification effect of surgery related products, cytochromes may be directly involved in the regulation of pro-or anti-emetic signals, for instance through the regeneration of serotonin by CYP2D6 (Yu et al., 2003; Kirchheiner et al., 2005).

Despite the development of a number of risk scores that aim to identify patients at risk of PONV and the availability of efficient preventive anti-emetic medication that can be targeted toward patients at risk, the overall incidence of PONV remains significant in clinical settings. Characterization of additional risk factors allowing an even better identification of patients at risk of PONV is therefore still highly clinically relevant. The aim of the current study is to validate the association between selected genetic polymorphisms and PONV in a cohort of surgical patients without antiemetic premedication.

## Material and Methods

### Clinical Trial

The study population was recruited in the frame of a multi-centric clinical study evaluating the efficacy of postoperative intra-venous administration of dexamethasone for the treatment of established PONV symptoms. Patients (>18 years old) undergoing elective surgery were recruited at Geneva University Hospitals (HUG), Lausanne University Hospital (CHUV), Réseau Hospitalier Neuchâtelois, Neuchâtel (RHNe) and Etablissements Hospitaliers du Nord Vaudois, Yverdon les Bains and Saint Loup (eHnv).

The clinical trial, described in detail elsewhere (Czarnetzki et al., in press), was approved by the Ethics Committee of Geneva (CER 11-213/NAC 11-076) and Swissmedic, the Swiss agency of medical products (SM2012DR2118) and registered on clinicaltrials.gov (NCT01975727). The study was conducted according to Swiss national law on clinical trials, following international ICH guidelines and the ethical principles of the Helsinki declaration. All patients were informed, agreed to participate and signed an informed consent form including questions regarding their choices for the handling of their genetic material.

Briefly, patients were included before undergoing surgery and those presenting an initial episode of nausea or vomiting in the first 24 h following waking after surgery, received the study treatment and were followed for another 24 h for recurrence of PONV symptoms, which consisted the primary end-point of the study. A rescue anti-emetic treatment was offered to patients showing persistent symptoms. Administration of study treatment was double-blinded and randomized between 4 groups receiving either 0 (placebo), 3, 6 or 12 mg dexamethasone.

### Inclusion/Exclusion Criteria

All patients had to have an American Society of Anesthesiology (ASA) status below IV.

Patient taking drugs with antiemetic properties (butyrophenones, 5-HT_3_ receptor antagonists, dexamethasone) or known emetogenic potency (for instance, l-Dopa, COMT inhibitors) were excluded. Similarly, patients with overt psychosis or taking antipsychotic treatment (such as, antidopaminergic drugs), or patients taking drugs interfering with platelet aggregation (such as aspirin or clopidogrel) in the week preceding the operation were excluded. Specific types of surgery increasing the risk of postoperative bleeding such tonsillectomy, as well as interventions or patients requiring strict prevention of postoperative vomiting were not taken in considerations. Other exclusion criteria included renal of hepatic dysfunction, gastrointestinal ulcer, systemic or local infections as well as the need for prolonged postoperative intubation or use of a gastric tube. For female participants of child-bearing age a negative pregnancy test was mandatory.

Beside prophylactic antiemetic and the exceptions mentioned above, there was no restriction on premedication, and compounds for anesthesia compound and postoperative analgesia, including opioid administration.

### Clinical Data Collection and Dichotomization

The data collected during the study included: age (years), gender (male/female), active tobacco or cannabis smoking habits (yes/no), self-reported previous history of PONV (yes/no), type of surgery (gynecologic, orthopedic, visceral, ENT, neurologic or plastic), use of volatile anesthetics during the operation (yes/no) as well as intra and postoperative use of opioids (type, amount and route of administration). Opioid posology was divided into three categories: intraoperative, postoperative until time of first PONV episode or, for patients without PONV, until the end of the 24 h observation period. Perioperative opioid consumption was expressed as mg of oralmorphine equivalents using standard conversion tables (HUG, 2009; Toulouse, 2016).

In case of patients undergoing gender re-assignment surgery (5 over 601 participants), final sex was considered as most relevant due to the potential effect of the hormonal status on PONV occurrence, considering that all genetic determinants explored were located on autosomal chromosomes.

All risk predictors were dichotomized according to their predicted influence on PONV (1 = for increased risk, 0 = no increase of risk). Age <50 years old, female sex, previous PONV history, visceral or gynecological surgery and use of volatile anesthesia, were considered risk factors, while tobacco and cannabis smoking were expected to be protective. Dichotomization for opioid consumption was based on median consumption with a median [IQR] consumption of 19 mg [6–46] mg morphine equivalent and thus 0 for values ≤19 mg and 1 for values above 19 mg morphine equivalent.

### DNA Extraction

The DNA purification and concentration at 50 ng/µL was performed in the laboratory for clinical pharmacology of Geneva University Hospitals using the MagJET Whole Blood Genomic DNA kit (ThermoFisher Scientific). DNA quantification was performed with the Qubit dsDNA BR Assay (ThermoFischer Scientific).

### Genotyping

Polymorphisms were tested on a QuantStudio 12K instrument using a custom OpenArray^®^ panel (ThermoFisher Scientific) operated by GenePredictis^®^ SA. All selected SNPs were chosen based on literature data suggesting direct correlation with nausea and vomiting in different contexts (PONV, CINV, pregnancy) or functional implication in metabolic pathway potentially related to the process. The full list is found in Table 3 for isolated SNPs and Supplementary Data S1 for polymorphisms used for cytochrome activity prediction.

The cytochrome *CYP2D6* copy number was determined in triplicate on a 7900HT Fast Real-Time PCR System (Applied Biosystems™) at the iGE3 Genomics Platform of the University of Geneva using TaqMan^®^ probes located within the exon 9 (Hs00010001_cn from ThermoFischer Scientific) and RNase P as reference. The study population was assumed to be mainly of European descend.

### Quality Control and Genotype Assignment

Raw data from the OpenArray^®^ and Copy Number Assay were treated using the TaqMan^®^ Genotyper™ and CopyCaller™ software (ThermoFischer Scientific) respectively. The Hardy-Weinberg equilibrium is respected for all SNP except *rs1065852* and *rs3755468* (cases, *p* < 0.001).

Validated calls were further processed using the Alleletyper™ software (ThermoFisher Scientific) to assign individual genotypes. When available, the star allele nomenclature from the Pharmacogene Variation Consortium (PharmVar, 2021) was used, otherwise the genotypes were reported as single nucleotide polymorphisms. CYP2D6 activity score was calculated following Gaedigk et al. (2008). CYP1A2, CYP2B6 and CYP2C19 activities were calculated on the same model as CYP2D6 with: *1A/*1L = 1, *1F = 2 and *1C = 0 for CYP1A2; *1/*9 = 1, *4/*22 = 2 and *5/*6 = 0 for CYP2B6 and *1 = 1, *17 = 2 and *2/*3 = 0 for CYP2C19. CYP3A activities were predicted following the most recent studies on tacrolimus dose adjustment (Elens et al., 2011) with one category increment for CYP3A7 activation in adults (*1C allele) (Burk et al., 2002). For CYP3A, extensive metabolizers (EM) were given an activity score of 2, intermediate metabolizers (IM) a score of 1 and poor metabolizers (PM) a score of 0. Finally, CYP2C9 activity was expressed as a sum of the number of alleles with reduced activities (Van Booven et al., 2010). When mentionned, CYP1A2 induction by smoke was taken into account by multiplying the activity score by 1.5× for smokers as previously described (Lesche et al., 2020).

### Statistical Analysis

Descriptive statistics were carried out with R v3.5.1 and Microsoft^®^ Office Excel version 2007. Logistic regression analysis according to Eq. 1 were performed using PLINK v1.07 (Purcell et al., 2007) for single nucleotide polymorphisms or R v3.5.1 for predicted cytochrome activities. Dichotomized values were used for co-variables. Cytochrome activities were considered as continuous values while single nucleotide polymorphisms were considered as ordinal variable. An additive genetic model was applied.

$$\begin{array}{l} Log\left(\frac{p}{1-p}\right)=\beta_{0}+\beta_{1}\mathrm{Gender}+\beta_{2}\mathrm{Age}+\beta_{4}\mathrm{Smoking}\\ +\beta_{5}\mathrm{Cannabis}+\beta_{3}\mathrm{PONVhistory}\\ +\beta_{8}\mathrm{SurgeryType}+\beta_{6}\mathrm{VolatileAnesthetics}\\ +\beta_{7}\mathrm{HighOpioid}+\beta_{9}\mathrm{GeneticMarker} \end{array}$$ (1)

Where p = probability of PONV occurence or recurrence.

Haplotypes blocks and corresponding correlations were determined in PLINK v1.07 based on a linkage disequilibrium analysis (Purcell et al., 2007) and were illustrated with Haploview (Barrett et al., 2005).

Statistic tests were considered significant if the *p*-value < 0.05 and correction for multiple testing was applied unless otherwise specified.

For modeling, PONV risk scores were calculated according to the simplified Apfel score (Apfel et al., 1999) and model parameters together with NNG according to Tonk and al. (2017). Receiver operating characteristic (ROC) curve analyses were performed online using the EasyROC tool (Goksuluk et al., 2016), available at http://www.biosoft.hacettepe.edu.tr/easyROC/.

## Results

### Study Population Characteristics and Genetic Integrity

Of the 803 study participants, 632 agreed to the genetic blood analysis, Nineteen of them were excluded due to incompleteness of their data record, leaving a preliminary sample pool of 613 patients.

The OpenArray^®^ technology was used to assess the genotype of 60 different single nucleotide polymorphisms (SNPs). Following quality control of the genotyping experiment, a total of 601 samples and 59 SNPs were cleared for final analyses. Both the mean sample genotyping call rate (CR) and the mean SNP call rate for the OpenArray^®^ experiment were >99% (99.35 vs. 99.41% CR respectively).

The characteristics of the final population is presented in Table 1. From the 601 participants, 264 (43.9%) suffered from PONV in the 24 h following their awakening from surgery. Of those, 229 patients (86.7%) were followed, and 157 (68.6%) of them presented a second episode of PONV. Administration of study treatment did not show any benefit on recurrence at neither dose (*p* > 0.1 whatever the model) compared with placebo (Czarnetzki et al., in press), leading to an early termination of the clinical trial for futility purposes.

**TABLE 1:** Population characteristics.

	All	With initial PONV	Without PONV	PONV patients with follow-up	With recurrence	Without recurrence
Total population (Nbr.)	601	264	337	229	157	72
Female (%)	52.6	70.8	38.3	73.8	73.2	75.0
<50 years (%)	57.7	60.2	55.8	62.0	63.7	58.3
Nonsmoking (%)	68.1	71.2	65.6	72.1	75.2	65.3
No cannabis (%)	93.7	94.7	92.9	95.2	95.5	94.4
With PONV history (%)	23.8	33.7	16.0	34.9	38.2	27.8
Visceral or gynecological surgery (%)	40.8	37.5	43.3	38.0	35.7	43.1
With volatile anesthesia (%)	87.5	90.2	85.5	91.7	91.7	91.7
With high opioid (%)	48.4	47.0	49.6	48.0	40.1	65.3

The minor allele frequencies (MAF) observed in the cohort are consistent with those of a standard population of European descent (according to the Ensembl and GnomAD repositories (Karczewski et al., 2020; Howe et al., 2021)) and similar between case and controls (Table 2, Supplementary Data S1, S2). Cytochrome P450 (CYP) enzymatic activities are often affected by combinations of genetic polymorphisms. Use of prediction tools reflecting the overall genetic impact of a set of mutations on protein activity show a resulting metabolizer profile for the study population that is consistent with the expected distribution in the general European population (Supplementary Data S3).

**TABLE 2:** Correlation between PONV and selected single nucleotide polymorphisms.

Gene	SNP ID	Chr	Major allele	Minor allele	MAF EUR^a^	MAF study	PONV Occurrence			PONV recurrence		
							OR	95% CI	P-value^b^	OR	95% CI	P-value^b^
*COMT*	*rs4680*	22	G (VAL)	A (MET)	0.50	0.47	1.09	0.85–1.41	0.493	0.92	0.60–1.41	0.693
	*rs4633*	22	C	T	0.50	0.47	1.06	0.83–1.37	0.629	0.93	0.61–1.43	0.745
	*rs165722*	22	C	T	0.50	0.48	1.09	0.85–1.41	0.492	0.95	0.62–1.45	0.800
	*rs6269*	22	A	G	0.41	0.43	0.91	0.71–1.18	0.484	0.95	0.62–1.45	0.801
	*rs4818*	22	C	G	0.40	0.41	0.90	0.70–1.17	0.434	0.89	0.58–1.37	0.594
*CHRM3*	*rs2165870*	1	G	A	0.34	0.34	0.97	0.75–1.26	0.845	0.97	0.63–1.48	0.878
	*rs10802789*	1	C	T	0.44	0.43	0.93	0.72–1.19	0.539	1.05	0.71–1.57	0.797
	*rs685550*	1	A	G	0.24	0.26	1.14	0.85–1.52	0.375	1.25	0.76–2.05	0.371
*HTR1A*	*rs6295*	5	C	G	0.46	0.49	0.89	0.69–1.14	0.360	0.88	0.59–1.32	0.550
*HTR2A*	*rs6313*	13	G	A	0.44	0.45	0.82	0.64–1.05	0.115	0.62	0.41–0.93	0.022*
*HTR3A*	*rs10160548*	11	T	G	0.33	0.39	0.97	0.76–1.24	0.809	1.24	0.81–1.81	0.321
	*rs1985242*	11	T	A	0.32	0.34	0.80	0.61–1.04	0.098	0.94	0.60–1.46	0.772
	*rs1176713*	11	A	G	0.21	0.25	0.79	0.59–1.04	0.097	0.93	0.58–1.51	0.782
*HTR3B*	*rs1176744*	11	A (TYR)	C (SER)	0.31	0.30	0.76	0.58–1.00	0.051	1.10	0.68–1.78	0.700
	*rs3758987*	11	T	C	0.29	0.27	0.73	0.55–0.97	0.033*	1.05	0.64–1.73	0.838
	*rs1672717*	11	A	G	0.42	0.35	1.45	1.12–1.89	0.005**	0.63	0.40–0.98	0.043*
	*rs3782025*	11	A	G	0.50	0.44	1.40	1.09–1.79	0.009**	0.66	0.43–1.00	0.052
	*rs76124337*	11	CA	—	0.40	0.33	1.47	1.12–1.91	0.005**	0.67	0.43–1.05	0.079
	*rs45460698*	11	AAG	—	0.07	0.14	0.84	0.58–1.21	0.352	0.92	0.51–1.66	0.772
*HTR3D*	*rs6443930*	3	G (GLY)	C (ALA)	0.47	0.49	1.01	0.79–1.30	0.916	1.26	0.83–1.92	0.275
*OPRM1*	*rs1799971*	6	A (ASN)	G (ASP)	0.16	0.15	0.88	0.62–1.25	0.474	0.98	0.55–1.76	0.949
*DRD2*	*rs1800497*	11	G	A	0.19	0.18	0.99	0.71–1.38	0.935	1.31	0.74–2.33	0.350
*TACR1*	*rs3755468*	2	C	T	0.45	0.44	0.77	0.60–0.98	0.032*	1.01	0.67–1.50	0.978
*FAAH*	*rs324420*	1	C (PRO)	A (THR)	0.21	0.20	1.15	0.85–1.56	0.364	0.63	0.38–1.05	0.076
*ABCB1*	*rs1128503*	7	G	A	0.42	0.40	0.96	0.74–1.24	0.750	0.91	0.60–1.40	0.679
	*rs1045642*	7	G	A	0.52	0.47	0.80	0.62–1.03	0.078	0.89	0.58–1.35	0.578
	*rs2032582* ^c^	7	C	A/T	0.41/0.02	0.38/0.02	0.91	0.70–1.18	0.472	1.06	0.70–1.61	0.782

a [59, 60].

b Signif. codes: 0 “***” 0.001 “**” 0.01 “*” 0.05.

c Triallelic SNP: P-value calculated in PLINK, in A vs C (MAF, for T allele = 0.016).

### Classical Risk Factor Evaluation

Recognized risk factors for PONV include: female gender, non-smoking status, a history of previous PONV, use of volatile anesthesia and perioperative opioid consumption. The role of patients’ age and the type of surgery itself remains less clear. The impact of each of those factors on the occurrence and recurrence of PONV our cohort are presented in Table 3.

**TABLE 3:** Logistic regression model based on dichotomous clinical factors.

	PONV occurrence			PONV recurrence		
	OR	95% CI	P-value^a^	OR	95% CI	P-value^a^
Intercept	—	—	5.01E-06***	—	—	0.483
Gender (0 = male, 1 = female)	4.11	2.85–5.96	6.02E-14***	0.92	0.50–1.96	0.800
Age group (0 ≥ 50, 1 < 50 years)	1.52	1.05–2.20	0.026*	1.35	0.68–2.31	0.332
Smoking (0 = yes, 1 = no)	1.35	0.91–2.01	0.140	1.56	0.85–3.21	0.193
Cannabis (0 = yes, 1 = no)	1.08	0.50–2.39	0.849	1.27	0.34–5.68	0.735
History of PONV (0 = no, 1 = yes)	2.38	1.56–3.65	6.87E-05***	1.86	0.95–3.56	0.067
Surgery (0 = other, 1 = visc, gyneco)	0.72	0.50–1.04	0.082	0.70	0.41–1.37	0.245
Volatile anesthetics (0 = no, 1 = yes)	2.88	1.66–5.12	2.30E-04***	0.92	0.29–2.67	0.886
High opioid (0 = no, 1 = yes)	0.85	0.59–1.22	0.378	0.35	0.21–0.71	6.99E-03**

a Signif. codes: 0 “***” 0.001 “**” 0.01 “*” 0.05.

Using dichotomized values to describe the cohort, we found that, female gender, history of previous PONV and use of volatile anesthetics were, as expected, significant risk factors for PONV occurrence. Advanced age showed a significant protective effect while tobacco smoking and cannabis consumption tended to be protective although the values did not reach statistical significance. It should however be taken in consideration that only 6.3% of the whole study population reported smoking cannabis. Although not statistically significant for the model, the trend observed for the type of surgery or level of opioid consumption did not follow the expected behavior. Interestingly, none of the factors known to influence the occurrence of PONV seemed to predict its reoccurrence, with even an apparent protective effect of high opioid consumption which seemed to reduce the risk with an odds ratio (OR) of 0.35 [95% confidence interval (CI): 0.21–0.71, *p*-value = 6.99E-03].

Using linear parameters for age (year) and opioid consumption (expressed as grams of morphine equivalent) did improve the overall model parameter for the prediction of PONV occurrence as well as the statistical significance of age and opioid consumption, however with an OR very close to 1.0 for both parameters (Supplementary Data S4). The lack of detailed timing of administration of opioids following surgery precluded further dissection of their specific role in the occurrence of PONV in this study.

Co-variable independence is one of the fundamental prerequisites of mathematical regression models. It is, however, clear in the current case that all variables in the model are not fully independent for each other. For instance, 71% of the participants with previous PONV history were women and 67% of the smokers were below 50 years of age. Although statistical independence test of each variable pair evaluated using Fisher exact test showed there were significant associations (*p*-values in Supplementary Data S5), a multicollinearity analysis showed that all variance inflation factors (vif) were <1.2 (see Supplementary Data S5), indicating that the observed parameter interdependence did not affect the overall model integrity. As patients were randomized and study treatment was administrated only after the first episode of PONV, dexamethasone administration had no influence on the occurrence of the initial PONV symptoms. However, while both administration and dosage of dexamethasone were thought to interfere with PONV recurrence, neither did show the intended effect (Czarnetzki et al., in press). Using the current regression model, we confirm that dexamethasone administration does not prevent reoccurrence of PONV in patients suffering from PONV symptoms nor does it influence the weight and the significance of the other model parameters of the logistic regression (Supplementary Data S6). Moreover, administration of dexamethasone, within the tested dose range, does not impact the weight or the significance of any of the genetic determinants tested (Supplementary Data S7).

### PONV Association With Single Nucleotide Polymorphism or Metabolic Enzyme Activities

The impact of each SNP or DME activity on occurrence or recurrence of PONV was assessed using logistic regression including the following covariates: age, gender, tobacco smoking, cannabis consumption, previous PONV history, use of volatile anesthetic and high perioperative opioid consumption. An additive genetic model, where each mutated allele contributes to the signal (i.e., AA = 0, AB = 1, BB = 2) was chosen for all polymorphisms tested. For metabolic enzymes, activity scores were considered as ordinal values. The association *p*-values and corresponding OR for first PONV occurrence and recurrence are presented in Table 2 for individual SNPs and Table 4 for cytochrome activities respectively. None of the associations reached statistical significance if a strict Bonferroni correction was applied (ie if *p* = 0.05/27—> corrected *p*-value = 0.002 or 2E-03).

**TABLE 4:** Predicted P450 cytochrome activities correlation with PONV¨.

Gene	Determinant	Chr	PONV Occurrence			PONV recurrence		
			OR	95% CI	P-value^1^	OR	95% CI	P-value^1^
*CYP2D6*	Activity score	22	1.02	0.79–1.32	0.862	0.88	0.56–1.39	0.594
*CYP3A*	Activity group	7	1.17	0.85–1.63	0.332	0.80	0.46–1.38	0.418
*CYP2C9*	Nbr. of reduced allele	10	0.78	0.56–1.09	0.147	1.42	0.80–2.63	0.249
*CYP2C19*	Activity score	10	1.09	0.88–1.35	0.415	1.00	0.70–1.42	0.987
*CYP1A2*	Activity score	15	1.17	0.90–1.52	0.239	**0.55**	**0.34–0.87**	0.012*
*CYP2B6*	Activity score	19	0.93	0.74–1.17	0.536	1.12	0.75–1.66	0.578

a Signif. codes: 0 “***” 0.001 “**” 0.01 “*” 0.05.

Four of the five polymorphisms tested showing an association with the occurrence of PONV map to the serotonin receptor 3B encoding gene (*HTR3B*). In addition, we found an association between the *rs3755468* mutation in *TARC1* and PONV occurrence, as well as between the *HTR2A rs6313* mutation and PONV recurrence (Table 2). Regarding predicted cytochrome activities, our results suggest an involvement of the highly inducible CYP1A2 enzyme in the recurrence of PONV [OR 0.55 (95% CI 0.34–0.87), *p*-value = 0.012]. None of the other predicted CYP activities seemed to significantly influence the occurrence or recurrence of PONV in our cohort (Table 4).

There was no effect of dexamethasone treatment at either dose nor globally on the results presented in Tables 2, 4 (Supplementary Data S7). As different genetic factors are likely to act on PONV through various mechanisms, we considered the influence of each genetic factor on the impact of classical risk determinants of PONV by looking for confounding. A systematic analysis of the influence of each polymorphism on the estimates of significant co-variable revealed only 4 cases of weak confounding effect (Supplementary Data S8).

Interestingly, two of the *HTR3B* polymorphism had an effect on the age association with PONV occurrence. Indeed, age segregation for heterozygous carriers of the *rs3782025* or *rs76124337* mutations reveals a clear risk difference. This effect, while already visible in the whole cohort for *rs76124337*, was highly predominant in women (Supplementary Data S9). Interestingly, both serotonin signaling and PONV risk are believed to be sex and age dependent (Yonezawa et al., 1989; Meltzer et al., 1998; Laugsand et al., 2011), further underlying the involvment of variations in brain serotonin homeostasis in PONV.

### The *HTR3B* Haplotype Associated With the Most Severe PONV Risk is Present in One Third of the Patients

Serotonin signaling through type 3 receptors is known to play an important role in emesis. The strong association observed between three of the *HTR3B* SNPs and PONV confirms previous reports (Rueffert et al., 2009; Ma et al., 2013; Yan et al., 2021). Moreover, the weaker associations found between the remaining *HTR3B* and *HTR3A* related SNPs and PONV occurrence, and to a lesser extend recurrence, further supports the observed link between nausea and vomiting and type 3 serotonin receptors.

The logistic regression analysis was based on an additive model where the presence of each additional allele contributes independently to the phenotype. Testing of the significant associations between *HTR3B*-related SNPs and PONV occurrence using dominant or recessive genetic models for the minor alleles, shows that the best description of the association for *rs3782025* is additive. Additive and dominant models are nearly equivalent for *rs76124337* and *rs1672717*, while a dominant model is more favorable for *rs3758987*. It is noteworthy that the association between *rs3782025* remained significant whatever the model used (Supplementary Data S10).

**FIGURE 1:**
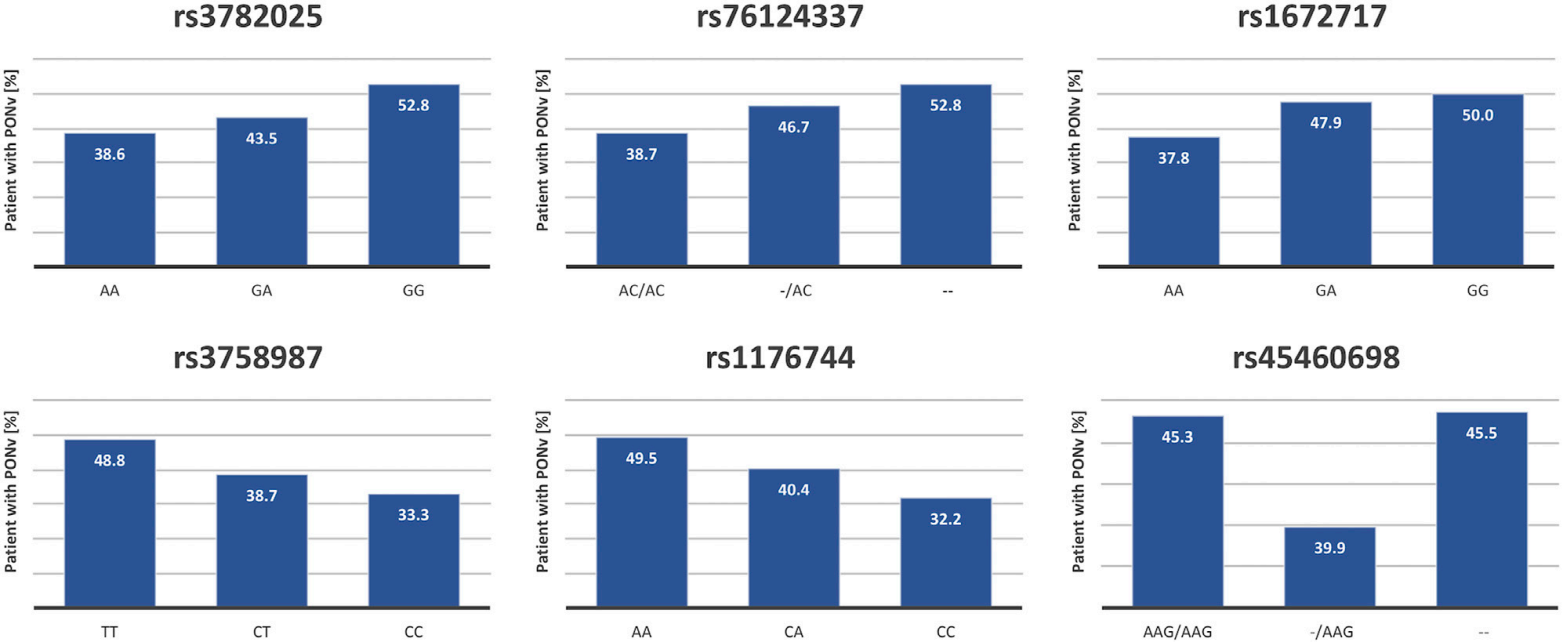
Correlation between *HTR_3B_* polymorphisms and PONV occurrence. Five of the six *HTR_3B_* polymorphisms tested in the screen were significantly correlated with the risk of PONV occurrence. The figure shows, for each of those SNPs, the proportion (%) of patients suffering from PONV in each genotypic category. The homozygous genotype corresponding to the major allele is always depicted on the left.

**FIGURE 2:**
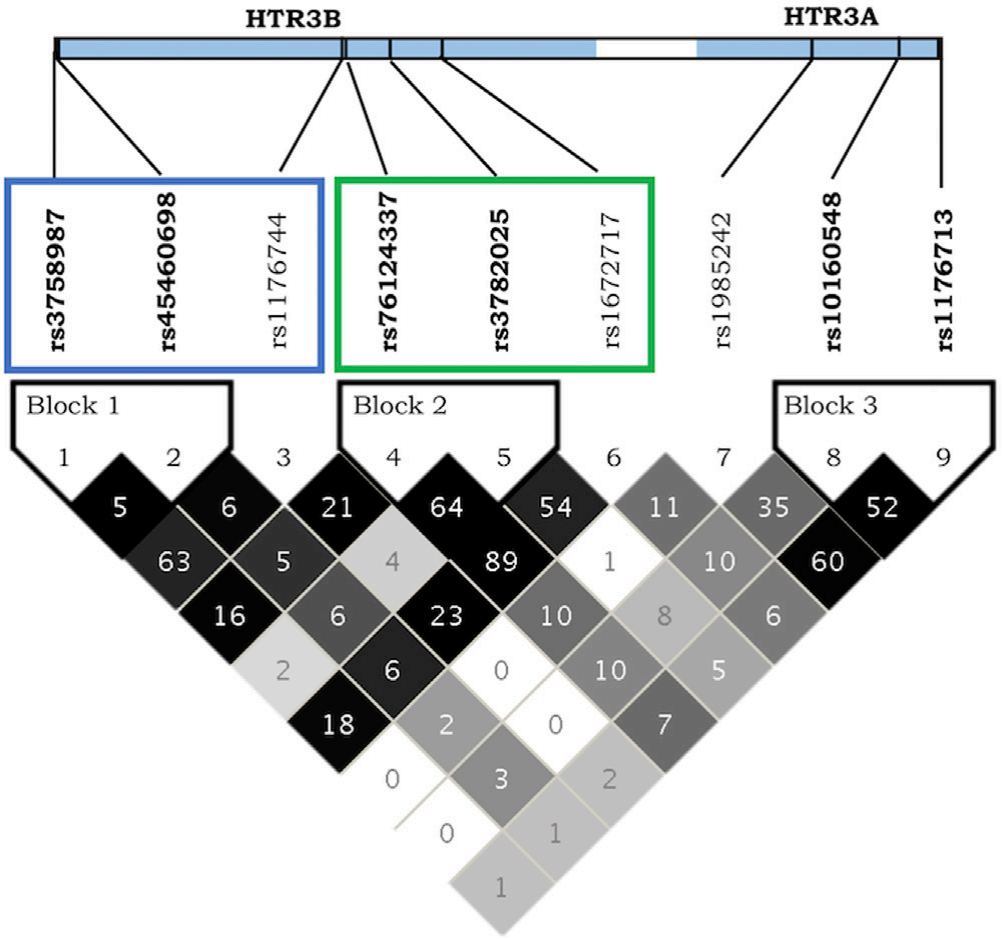
LD plot for *HTR3A* and *HTR3B* receptors on Chromosome 11. The shading of the haplotype boxes indicates the magnitude of LD coefficient D’, with darker color indicating stronger LD. 100-fold pairwise *r* ^2^ squared numbers are displayed. Blue rectangle highlights *HTR3B* mutations with positive influences on PONV prediction while the green box indicates *HTR3B* mutation with increased risks of PONV. Produced with Haploview (Barrett et al., 2005).

Interestingly, not all *HTR3B* mutations showed the same trend of association with PONV: some alleles had a protective effect while others were clear risk factors suggesting the presence of two blocks of mutations (Figure 1). A linkage disequilibrium analysis detected three haplotype blocks on chromosome 11, two within *HTR3B* (*rs3758987* and *rs45460698*; *rs76124337* and *rs3782025*) and one within *HTR3A* (*rs10160548* and *rs1176713*) (Figure 2). As highlighted in Figure 2, the two *HTR3B* variant blocks identified by the haplotype analysis corresponded to the positive and negative PONV prediction trends observed for *HTR3B* mutations.

Association between PONV and the haplotypes blocks on chromosome 11 confirms the predominance of the second block composed of *rs76124337* and *rs3782025* for the prediction of PONV (Table 5) with the–G genotype increasing the risk of PONV by a factor of 1.49. Similarly, the combination of all six mutations of *HTR3B* together also highlights the importance of the same SNPs for prediction of PONV occurrence. Interestingly the predicted “wild-type” or reference haplotype, corresponding to the major alleles of each independent SNPs was present in only 19% of the population, while the worst combination in terms of PONV occurrence, with an odd ratio of 1.5, was found in 32% of the study population.

**TABLE 5:** Correlations between serotonin receptor haplotypes and PONV occurrence.

Gene	Haplotype Block^a^	Nbr SNP	Population coverage [%]	Haplotype	Frequency	PONV Occurrence		
						OR	STAT	P-value^b^
*HTR3B*	1	2	100	T-	0.14	0.84	0.86	0.355
				CAAG	0.26	0.74	4.27	0.039*
				TAAG (wt)	0.60	1.39	6.33	0.012*
*HTR3B*	2	2	100	-G	0.33	1.47	7.93	0.005**
				ACG	0.11	1.01	1.52E-3	0.969
				ACA (wt)	0.56	0.71	7.12	0.008**
*HTR3A*	3	2	100	GG	0.25	0.78	3.00	0.083
				GA	0.14	1.41	3.57	0.059
				TA (wt)	0.62	1.03	0.04	0.840
*HTR3B*	All	6	92	TAAGAG-G	0.32	1.49	8.23	0.004**
				TAAGAAACG	0.02	0.95	1.26E-2	0.911
				CAAGCGACA	0.07	0.79	0.76	0.382
				TAAGCGACA	0.01	1.27	0.20	0.656
				CAAGCAACA	0.17	0.70	3.72	0.054
				TAAGCAACA	0.05	0.83	0.35	0.555
				T-AAACA	0.13	0.79	1.42	0.233
				TAAGAAACA (wt)	0.19	0.99	8.36E-3	0.927

a Block 1: HTR3B rs375987 + rs45460698.

Block 2 HTR3B rs76124337 + rs3782025.

Block 3 HTR3A rs10160548 + rs1176713.

All rs375987/rs45460698/rs1176744/rs3782025/rs76124337/rs1672717.

b Signif. codes: 0 “***” 0.001 “**” 0.01 “*” 0.05.

Taken together, our genetic analysis has identified two single nucleotide polymorphisms located in the open reading frame of type 3B serotonin receptor on chromosome 11 as the most important genetic predictor of PONV in our cohort.

### Serotonin Type 3B Receptor Mutation Versus Pharmacological Modulation of Serotonin

As serotonin is known to play an important role in emesis and as we were able to involve several SNPs related to type 3B serotonin receptors in the occurrence of PONV, we examined the effect of *HTR3B* genotype in patients having received tramadol as part of their postoperative analgesic treatment. Tramadol is an opioid with additional serotonin and norepinephrine reuptake inhibitor properties, thus promoting an increase in the synaptic cleft serotonin concentrations.

**FIGURE 3:**
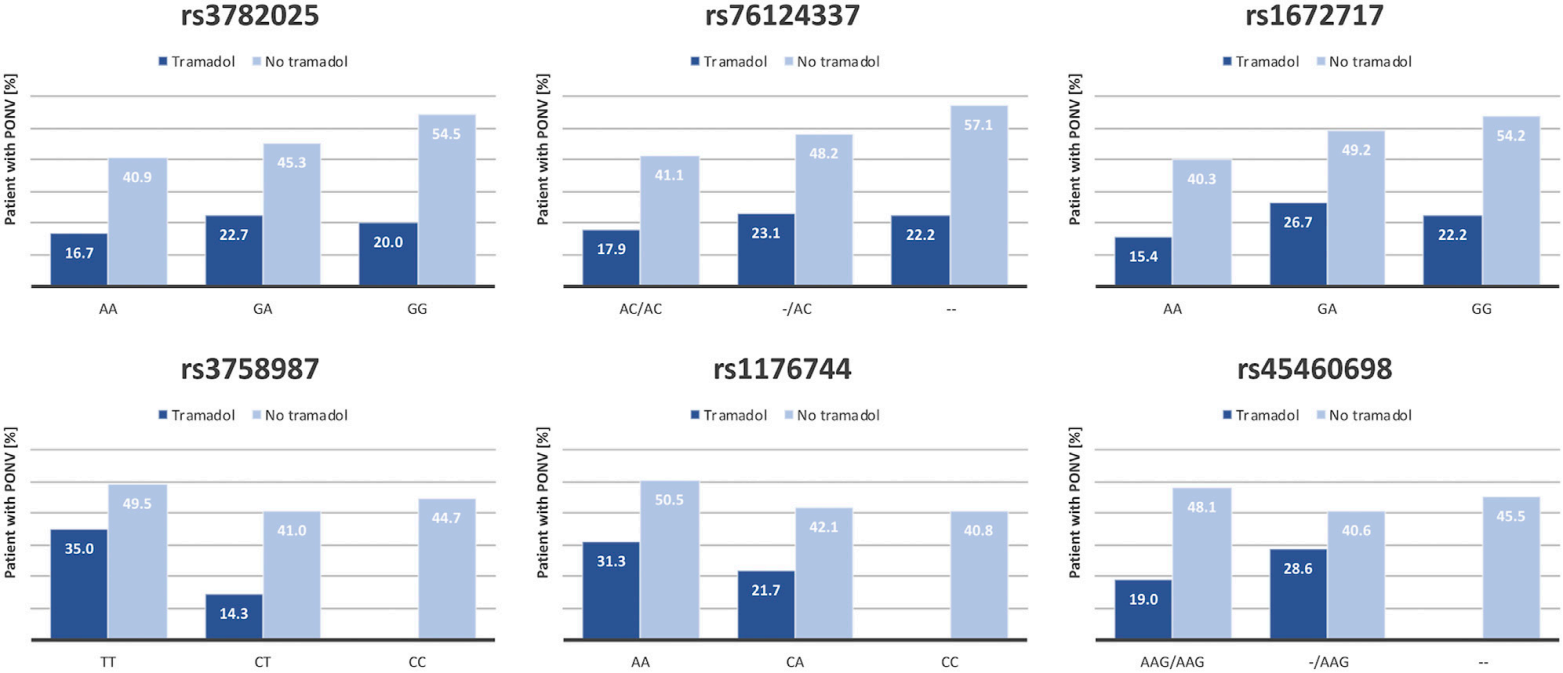
Effect of tramadol on association between *HTR3B*-related polymorphisms and PONV occurrence. Tramadol is a selective serotonin reuptake inhibitor that could influence *5-HTR_3_* receptor function and thus modulate the effect of *HTR3B* polymorphisms on PONV occurrence. The figure shows the proportion (%) of patients suffering from PONV in function of tramadol administration in each genotypic category for each of the six *HTR3B* SNP tested.

In the current study, 50 patients (8.3% of the study population) received tramadol as part of their postoperative analgesia. Despite known emetic properties of this opioid, only 10 (20%) of the patients under tramadol experienced PONV, versus 43.9% in the total cohort suggesting a selection bias. However, when comparing the effect of the *HTR3B* variant on PONV occurrence in patients under tramadol with patients without, two different tendencies became apparent (Figure 3). First, administration of tramadol seems to limit the negative impact of *rs3782025*, *rs76124337* and *rs1672717* variants. Second, the risk of PONV in patients with double mutation at position *rs3758987* or *rs1176744* receiving tramadol seems to be considerably diminished. Actually, the protective effect of those two last mutations relied to some extend on patients receiving tramadol (Supplementary Data S11). The current clinical study was not designed to address the impact of tramadol on PONV and the available data clearly lack the appropriate power for formal statistical analysis on this point.

### Incorporation of *HTR3B* Genotyping Information can Improve Prediction Capacity of the Classical Risk Scores

The simplified Apfel score is a tool for PONV prediction (Gan et al., 2020). In this score, the risk category of each patient is obtained by adding one unit for each of the following risk factors: female gender, non-smoking status, history of previous PONV and opioid consumption, and each risk category is associated with an increasing probability of suffering from PONV (Apfel et al., 1999).

**FIGURE 4:**
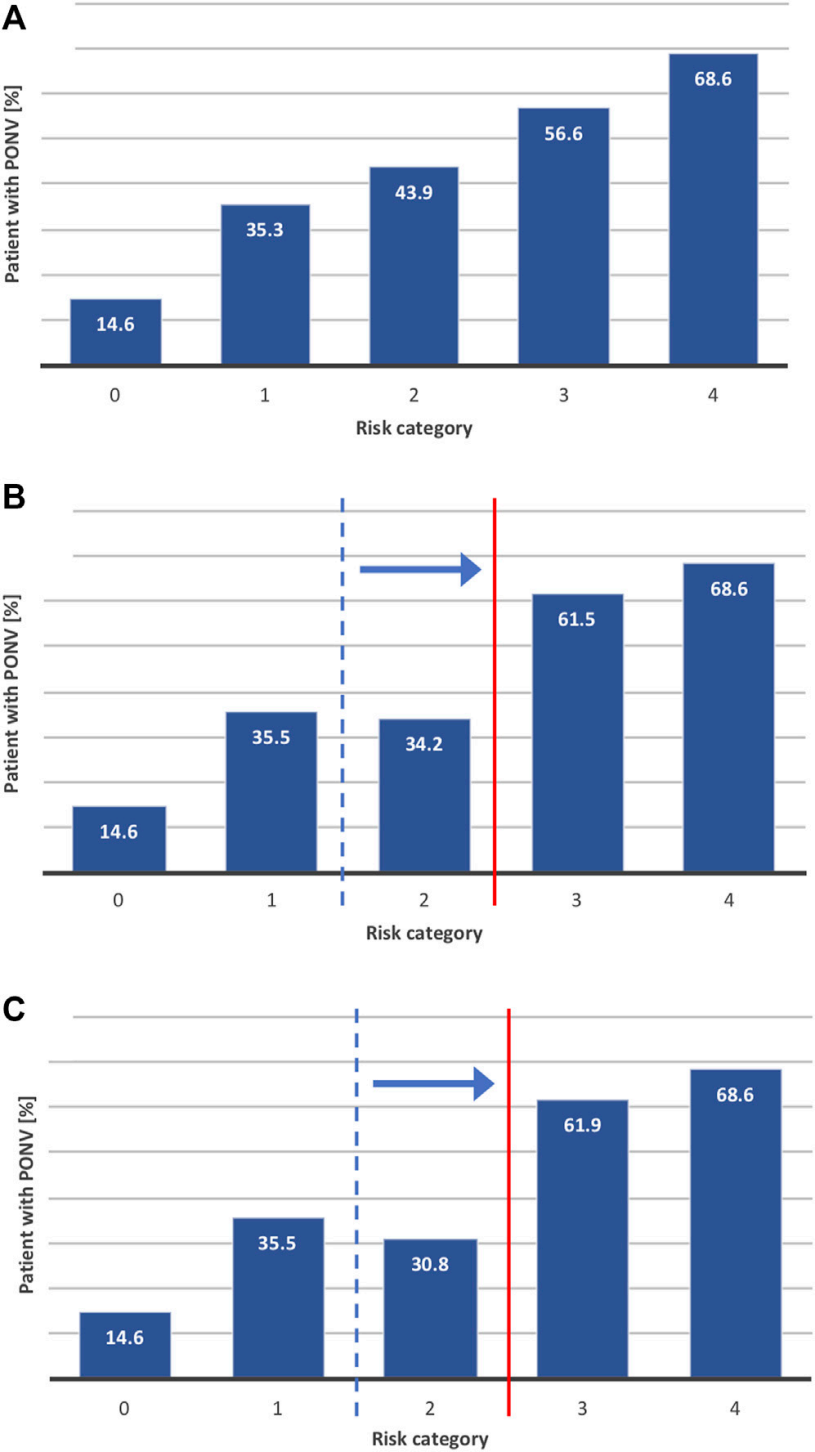
Integration of *HTR_3B_ rs3782025* genetic information in prediction score modeling. We tested three different models to evaluate the potential impact of integrating *HTR_3B_ rs3782025* genotype in the prediction score. The figures shows the proportion (%) of patients predicted to suffer from PONV in each risk category of **(A)** classic risk score (Apfel score) without genetic information **(B)** Apfel score + *rs3782025* genotyping for women below 50 years of age in Apfel score category 2 **(C)** Apfel score + *rs3782025* genotyping for all women in Apfel score category 2.

Application of the Apfel score to our study population followed the expected risk trend with a near linear increase of PONV risk from one category to the next as shown in Figure 4A. According to standard nomenclature, category 0 and 1 are generally considered at low-risk of PONV, category 2 patients are at medium risk while categories 3 and 4 regroup high-risk patients (Gan et al., 2020). While in our study population the *HTR3B* SNP *rs3782025* was the most relevant polymorphism for the prediction of PONV, the strength of the association of this parameter did not support a cost-effective genotyping in the whole cohort. However, considering that the classification of low and high-risk patients is unlikely to change due to genotype, medium-risk patients are the ones that are most likely to benefit from genotyping. In addition, the confounder analysis described above and shown in Supplementary Data S9, highlighted the predominance of the impact of *rs3782025* in younger women. Thus, we developed two models focalizing on medium risk (Apfel category 2) female patients to evaluate the clinical impact of genotyping. In Model 1, only category 2 female patients below 50 years old were genotyped while in Model 2 the genotype of all category 2 female patients was taken into consideration. In all cases, *rs3782025* genotypic risk was assigned according to the following rules: A/A = 0 and G/G = 1 in all cases, while A/G = 0 if patient is > 50 years old and = 1 if younger.

As summarized in Table 6, the Model 1 group covered 80 of our 601 patients, with an average PONV risk of 64% before adjustment. Adding the genetic information at locus rs3782025 for this group resulted in accurate PONV prediction for 68% of the patients with an average PONV occurrence of 75% in the higher risk group and 44% in the lower risk group. Clinical relevance can be estimated by calculating the number needed to genotype (NNG), which is obtained by dividing the number needed to treat (NNT) by the frequency of the risk allele occurrence (Tonk et al., 2017). In our case, this represents the number of patients that needs to be genotyped for one to be classified into the proper risk group and thus to be likely to benefit from PONV prophylaxis. Selection of patients according to Model 1 results thus in an NNG of 5 if considering identification of patients which might benefit of a reinforced PONV prophylaxis versus an NNG of 9 to avoid administration of unnecessary anti-emetic treatment. Figure 4B shows the actual incidence of PONV in the genotype-adjusted Apfel risk score categories when Model 1 is applied to our study population. The results for Model 2 are relatively similar to Model 1, with 67% accurate prediction for 124 patients responding to the selection criteria and an improvement from 9 to 7 of the NNG for detection of lower risk patients. Graphical representation of the PONV risk for each category calculated according to Model 2 is shown in Figure 4C.

**TABLE 6:** Prediction model parameters.

	Model 1^a^	Model 2^b^
Nbr patients genotyped	80	124
% PONV in genotyped patents	64	58
Nbr of patients with genetic risk factor^c^	51	66
% PONV in patients with genetic risk factor	75	72
% PONV in patients without genetic risk factor	44	40
Sensitivity	0.75	0.67
Specificity	0.56	0.65
NNG for identification of patients with increased PONV risk	5	6
NNG for identification of patients with decreased PONV risk	9	7

a Model 1: rs3782025 genotype for category 2 women <50 years.

b Model 2: rs3782025 genotype for all category 2 women.

c rs3782025 G/G = 1; rs3782025 A/G = 1 for women <50 years, rs3782025 A/G = 0 for women >50 years.

Receiver operating characteristic (ROC) curve analysis presented in Supplementary data S12 shows a significant although modest increase in the area under the curve (AUC) for both models with respect to the classic score with an AUC_Model1_ = 0.648 [95% CI 0.538–0.759], *p* = 8.27E-03 and AUC_Model2_ = 0.668 [95% CI 0.584–0.753], *p* = 9.62E-05 for the subgroups of patients where the genotype is taken into account. If considering the whole cohort, the calculated AUC for the classical Apfel score of 0.633 is slightly improved to 0.660 [95% CI 0.617–0.702] using Model 1 and further to 0.665 [95% CI 0.623–0.708] using Model 2.

### Effect of Phase 1 CYP450 Predicted Metabolism on PONV Recurrence—A Role for CYP1A2?

**FIGURE 5:**
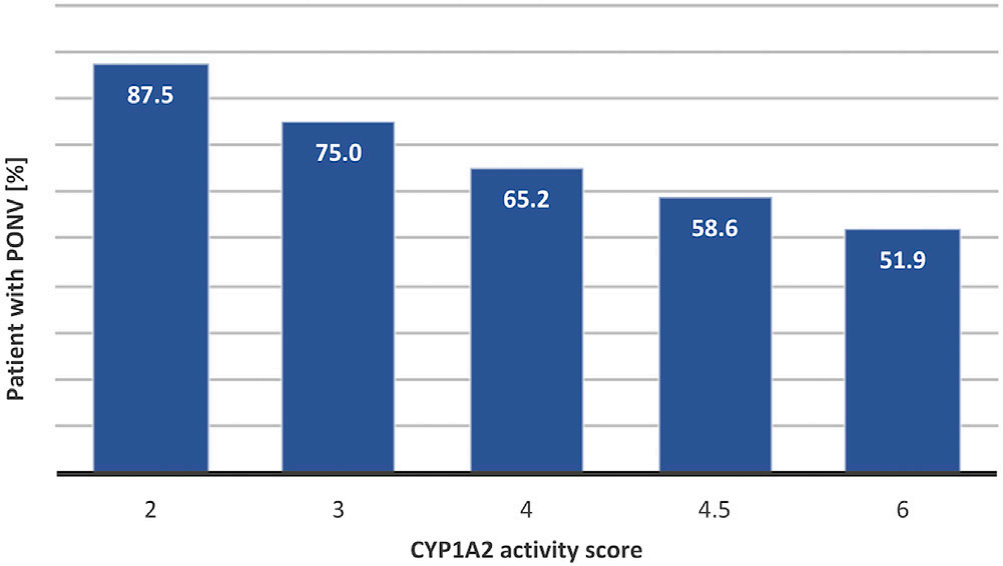
Correlation between CYP1A2 activity and levels of PONV. Predicted CYP1A2 activity scores have been calculated based on genotypic data taking into account activation due to smoking by multiplying the genotypically predicted activity score by 1.5× in presence of smoking (Lesche et al., 2020). The figures showy the proportion of patients experiencing PONV in each CYP1A2 activity category. The *R* ^2^ of the linear regression between score activity and PONV risk reaches 0.957.

CYP1A2 is a highly inducible cytochrome from the P450 family, with a large inter-individual variability. It is an abundant liver phase I enzyme as it represents >10% of all liver cytochromes P450, metabolizing a large number of substrates including drugs (clozapine), endogenous substrates (melatonin, steroids) and dietary products (caffeine). Both genetic and environmental factors are strongly influencing CYP1A2 activity, underscoring the difficulty in predicting its activity (Thorn et al., 2012). CYP1A2 is strongly induced by smoking. Our initial correlation between PONV recurrence and CYP1A2 activity did only take into account the genetics resulting in an OR of 0.55 (95% CI 0.34–0.87, *p*-value = 0.012). Adjusting the predicted CYP1A2 activity for smoking using the method developed by Lesche et al. (2020) resulted in a slightly improved correlation with an OR of 0.57 (95% CI 0.38–0.85, *p*-value = 0.006) (Figure 5).

CYP1A2 parallels the effect of smoking on the reduction of the risk of PONV and confounder analysis revealed that the two parameters are indeed interconnected. It has already been proposed that part of the protective effect of smoking toward PONV could be mediated by enhanced detoxification resulting from CYP1A2 and CYP2E1 activation (Sweeney, 2002).

## Discussion

The current genetic study first examined the impact of 28 individual single nucleotide polymorphisms (SNPs) located around 13 genes as well as the activity of six liver cytochromes (CYP) on the occurrence of PONV in a cohort of 601 patients without anti-emetic prophylaxis followed during the first 24 h after surgery. The overall PONV incidence in the cohort was 44%. We detected five polymorphisms that were significantly associated with PONV occurrence, one located in the promoter of the neurokinin receptor *TACR1* and the four others around the type 3B serotonin receptor gene (*HTR3B*). Further statistically significant risk factors in our cohort were female gender, history of PONV, and the use of volatile anesthetics.

5-HTR_3_ receptors are the only ligand-gated ion channel receptors activated by serotonin (Walstab et al., 2010). They are expressed throughout the brain as well as on gastrointestinal tract vagal afferents (Walstab et al., 2010). In the central nervous system, 5-HT_3_ receptors mediate fast excitatory synaptic transmission in response to serotonin, promoting neurotransmitter release (Walstab et al., 2010; Rees et al., 2017). HTR3B functions as a penta-heteromeric complex together with HTR3A (GeneCards, 2021). Polymorphisms in both *HTR3A* and *HTR3B* have previously been associated with early onset PONV (<6 h postoperatively) (Rueffert et al., 2009) as well as a response to antiemetic treatment in pregnant women (Lehmann et al., 2013) and CINV (Tremblay et al., 2003; Kaiser et al., 2004). As shown in Figure 1, we identified two blocks of mutations with opposite effects on PONV prediction. Additional analyses confirmed the linkage disequilibrium within each of the two mutation blocks and revealed that the haplotype corresponding to the highest risk group in terms of PONV occurrence was present in 32% of the study population and was best represented by *rs3782025*.

From the 6 *HTR3B* SNPs included in this screen, only *rs1176744* is located within an exon and results in a missense mutation (Y129S) known to result in a global increase receptor activity, with longer channel opening time, slower desensitization and deactivation kinetics (Krzywkowski et al., 2008; Walstab et al., 2008). This increase in activity tends to have a protective effect toward PONV occurrence which is in concordance with previous observations (Laugsand et al., 2011). The impact of the other identified mutations on 5-HTR_3_ receptor activity has, to our knowledge, not been described, but it is tempting to speculate on a reduced activity for the other block of mutations. The last *HTR3B* polymorphism tested (*rs45460698*), whose impact on CINV is controversial (Tremblay et al., 2003; Perwitasari et al., 2011), did not show any significant trend in the present study.

The confounder analysis suggesting a relationship between *HTR3B rs3782025* and *rs76124337* and patient age and gender is noteworthy. For both mutations, our results suggest a shift from a recessive to a dominant genetic model with age in female patients, while the mutated alleles have little impact in men of either age group. Several studies have suggested differences in serotonin signaling with age and gender, possibly linked, at least in part, to sex hormone signaling in women of child-bearing potential (Hernández-Hernández et al., 2019; Hudon Thibeault et al., 2019; Pottoo et al., 2019). This observation is in line with previous reports showing no gender difference in PONV occurrence in children up to puberty, after which a higher incidence is observed in girls than boys (Heyland et al., 1997; Eberhart et al., 2004; Ames and Machovec, 2020). However, although an age below 50–55 years is a recurring PONV risk factor, none of the studies reporting age as a significant factor (Sinclair et al., 1999; Junger et al., 2001; Leslie et al., 2008), performed sex specific analysis for different age groups in an adult population. Our data showed no modification in the gender related odds ratios for PONV in function of age. However, a number of factors other than hormones could play a role in the gender susceptibility toward PONV in older adults, including a global decrease in serotonin neurotransmission (Meltzer et al., 1998).

Current guidelines recommend the administration of 1–2 antiemetic agents for patients presenting up to two risk factors versus 3–4 agents for patients with more than two risk factors (Gan et al., 2020). The difficulty is to balance the need for antiemetic prophylaxis with the risk of adverse effects and increased cost, resulting from poly medication. Indeed, none of the available anti-emetic treatments is devoid of potential adverse effects and the concomitant administration of multiple drugs increases the risk of interaction and occurrence of adverse reactions (Alomar, 2014; Bennett and Sofat, 2020). Although the cost of genetic testing is decreasing rapidly, currently, genotyping all surgery patients is unlikely to be cost-effective. By focusing on the group of medium risk patients that would benefit most from it, our model shows that genotyping might improve the management of PONV in this population. Adding the genetic factor to prediction scores could allow evidence-based decision making regarding antiemetic prophylaxis in those patients, both offering an increased protection to patients at risk, and decreasing unnecessary exposure to potentially harmful treatments. Moreover, knowledge about 5-HTR_3_ receptor polymorphism might also be used to help predict the efficacy of 5-HTR_3_ antagonists, further enhancing the benefit of the genetic testing.

*TACR1* encodes a G-protein coupled receptor activated by neurokinin-1 (aka substance P). Neurokinin-1 is a neuropeptide involved in pain signaling and inflammation as well as emesis (Vilisaar and Arsenescu, 2016; Schank and Heilig, 2017). TACR1 receptor antagonists have been successfully used as antiemetic medications in CINV and PONV (Aziz, 2012; Razvi et al., 2019; Gan et al., 2020). The *rs3755468* C > T polymorphism in the *TACR1* promoter has previously been associated with reduced PONV occurrence and gender susceptibility in a Japanese population (Hayase et al., 2015). As the mutation is found in a predicted ERE (estrogen response element), it was hypothesized that this SNP could participate in the gender susceptibility toward PONV (Hayase et al., 2015). While we saw a similar protective effect of the *rs3755468* mutation in our cohort, this effect was approaching significance in men (OR 0.69, 95% CI 0.47–1.01, *p*-value = 0.06) but not in women (OR = 0.83, 95% CI 0.61–1.14, *p*-value = 0.24) in the opposite of the previous study. It should be noted that there is no considerable MAF difference between European and Japanese populations at this position. Thus, while the protective effect of the *TACR1 rs3755468* T allele seems reproducible, the differences observed with the results from the Japanese study suggests that more work is needed to unravel the role of *TACR1 rs3755468* polymorphism in PONV.

While both serotonin and substance P are known to play an important role in the transduction of pro-emetic signals, a number of other neurotransmitters involved in PONV have been identified, either through the blocking action of anti-emetic treatments or genetic screening (Gan, 2007; Horn et al., 2014; Janicki and Sugino, 2014). The current study failed to replicate a number of previously described associations between PONV occurrence and clinically relevant polymorphisms affecting recognized nausea and vomiting signaling pathways, such as dopamine, acetylcholine, opioid or even serotonin signaling through other types of 5-HT receptors (Hornby, 2001; Aziz, 2012; Janicki and Sugino, 2014; Belkacemi and Darmani, 2020). A number of technical reasons might explain differences between our and other studies starting by different ethnic background, the absence of anti-emetic prophylaxis in the present study as well as a potential overlap sometimes observed between PONV and opioid-induced nausea and vomiting. Indeed, as opioid administration did not appear to be a significant risk factor in our cohort, genetic factors affecting pathways induced by opioids are unlikely to show significant associations. In addition, the important cross-talk between the different neurotransmitter signaling pathways in the central nervous system has to be taken into consideration. Indeed, both HTR3 and TACR1 are key modulators of synaptic signal transduction through release of neurotransmitter from pre- and post-synaptic neurons and are in turn regulated by other neurotransmitters (Adell et al., 2010; Rees et al., 2017; Schank and Heilig, 2017; Peters et al., 2021). Thus it is difficult to distinguish whether the action of any anti-emetic drug is direct or through modulation of other neurotransmitter pathways. Similarly, genetic variants resulting in physiological differences in neurotransmitter signal transmission are likely to have a broad impact on central nervous system organization and neuronal interconnection.

The second part of this work examined the influence of the selected genetic markers on the recurrence of PONV following administration of dexamethasone. Although the anti-emetic mechanism of action of dexamethasone remains unclear, it is, in the PONV setting, an effective prophylactic treatment when used alone or in combination with other anti-emetic medication (Chu et al., 2014; Gan et al., 2020). According to the study protocol, patient suffering from PONV during the first 24 h after surgery randomly received an intravenous dose of 0 (placebo control), 3, 6 or 12 mg of dexamethasone (Czarnetzki et al., in press). As the study treatment was administrated only after apparition of the first PONV symptoms, it was of no concern for the search for genetic determinants of PONV occurrence. PONV recurrence after treatment administration on the other hand, would have been expected to depend on both the pharmacokinetic and pharmacodynamic properties of dexamethasone and the inter-individual variability affecting these parameters. Interestingly, administration of dexamethasone to patients suffering from PONV did not show any benefit, resulting in premature termination of the study for futility purpose (Czarnetzki et al., in press). The reanalysis of the study data taking into account the genetic information, in addition to the previously assessed risk factors, failed to reveal any potential genetic bias that might have occluded a positive effect of dexamethasone for the treatment of established PONV. The lack of association between the study treatment and the primary outcome as well as the absence of confounding effects with any of the polymorphisms or DME activity level tested, suggest that the observed associations between PONV recurrence and the *HTR2A rs6313* polymorphism and CYP1A2 activity is a direct result from the surgery and anesthesia rather than dexamethasone administration.

There is little previous information on the involvement of *HTR2A rs6313* in PONV, although *rs6311*, which is in strong LD with *rs6313* (LD *r* ^2^ = 1.000, D’ = 1.000) has been involved in severe nausea resulting from the adverse effect of paroxetine treatment in Japanese patients suffering from depression (Kato et al., 2006). The protective effect of the A allele, seen here, mirrors the results of Kato et al. (Kato et al., 2006). The rs6313 polymorphism is located in exon 1 and results in a silent mutation at the Ser34 position of the *HTR2A* gene, a neuronal G-coupled serotonin receptor involved in signals transmission from the synaptic cleft via PLC and PLA_2_ activation (Yevtushenko and Reynolds, 2010; Masson et al., 2012; Iglesias et al., 2017). Although the *rs6313* SNP has been linked to a number of diseases including schizophrenia, depression and eating disorders, the associations remain weak and the impact of the mutation on receptor activity remains uncertain, with some studies suggesting a decreased expression and protein level for the G allele, while other failed to measure a difference (Polesskaya and Sokolov, 2002; Bray et al., 2004; Parsons et al., 2004; Norton and Owen, 2005). However, 5-HTR_2A_ receptors are intimately involved in the neurotransmitter crosstalk in the brain (Norton and Owen, 2005; Peters et al., 2021) and could be involved in transmission or modulation of pro-emetic signals.

It is important to note that CYP1A2 activity was not correlated with the risk of PONV occurrence but recurrence, decreasing the likelihood that the effect is due to a direct effect of faster turnover of anesthetic drugs. Indeed, a faster metabolism of propofol, which is an intravenous general anesthetic drug shown to be protective of PONV (Dinis-Oliveira, 2018), by CYP1A2 (Murayama et al., 2007), is not coherent with the observed protective effect of this cytochrome. Lidocaine, another known CYP1A2 substrate (Orlando et al., 2004), is a local anesthetic drug that was shown, when administrated intravenously, to decrease the need of postoperative analgesia and thus believed to protect against PONV by decreasing opioid consumption (Wang et al., 2019). While perioperative administration of opioids belongs to the classical risk factors of PONV, this effect was not observed in the current study. Moreover, we could not detect any confounding effect between CYP1A2 activity and opioid dosage (Chang and Kam, 1999; Faber et al., 2005). Although not demonstrated, an interplay between CYP1A2 activity and the serotoninergic system is possible. Indeed, CYP1A2 substrate specificity is quite broad and the enzyme is known to metabolize both ondansetron (Huddart et al., 2019) and melatonin (Skene et al., 2001). Ondansetron is a serotonin receptor antagonist sharing a similar structure with serotonin; melatonin a downstream product of the serotonin pathway. Thus, it is possible that a high basal level of CYP1A2 activity, either due to genetic polymorphism or a habit of smoking, could set a higher degree of tolerance against increased serotonin signaling.

There are several limitations to the current analyses. First, by selecting a limited number of polymorphisms from the literature, we might have missed undescribed associations as well as introduced a bias towards the most often studied genes in the context of PONV. Indeed, four of the five significant associations uncovered for PONV occurrence belong to the *HTR3B* gene, and are in linkage disequilibrium, meaning those associations are not independent from each other. Genome-wide studies offer the advantage of an unbiased selection and the possibility to uncover novel associations, however, the power of such association study relies heavily on a larger sample size (Tam et al., 2019). Secondly, the initial SNP selection included a number of assumptions such as the pro-emetic effect of opioids that we did not see in this cohort, thus decreasing the interest in polymorphisms affecting genes directly related to opioid signaling such as *COMT*, *OPRM1*, *ABCB1* or *FAAH* (Coulbault et al., 2006; Janicki and Sugino, 2014; Sadhasivam et al., 2015). Next, although inter-individual variability, drug metabolism and elimination of volatile anesthetics as well as other intraoperatively administrated drugs might have been expected to play a role, none of the cytochrome activities tested was significantly associated with the occurrence of PONV. This might, on one hand, be due to treatment heterogeneity occulting significant effects, and on the other hand be a consequence of predicting cytochrome phenotypic activity based solely on genetic information. Indeed, enzymatic activity is a result of a combination of genetic and environmental factors. Thus, while genetically-derived predictive activity of most cytochromes tested in the current study is widely used, discrepancy might be observed especially for highly inducible enzymes (Shah and Smith, 2015). Also, regarding the risk score model parameters it should be noted that the non-inclusion of patients having received anti-emetic prophylaxis might have biased our cohort toward lower risk patients and thus enhance the AUC value of our models. Finally, in absence of a more powerful tool, we used the Apfel score to predict the baseline risk of PONV in individual patients. This score is pragmatic and also widely used in clinical practice. However, its discrimination (ability to distinguish between patients with and without PONV) and calibration (agreement between observed and expected outcome frequencies) are limited (van den Bosch et al., 2005).

Future perspectives include the validation of the clinical impact of the risk model including genetic parameters in a prospective study. Moreover, investigations focusing on the effect of tramadol in combination with *HTR3B* mutations could provide valuable information on the molecular mechanism of the 5-HTR_3_ receptors and enhance our understanding of its role in PONV. It is indeed difficult to draw conclusions from the effect of tramadol on the *HTR3B* SNPs seen in the current study, both due to the limited number of patients having received the drug and the apparent lack of an emetogenic effect of tramadol in those who did. However, our preliminary observations suggest that administration of tramadol, which, like other selective serotonin receptor inhibitors, tend to increase serotonin levels in the extracellular space, might limit the negative impact of *rs3782025*, *rs76124337* and *rs1672717* variants and further diminish the risk of PONV in patients with a double mutation at position *rs3758987* or *rs1176744*. In a broader view, the next challenge facing genetic studies in the context of PONV will be to unravel the contribution of different SNP to the occurrence of PONV versus response to antiemetic treatments. Although large cohorts including administration of standardized antiemetic treatment will be necessary to reach statistical significance, understanding the interplay between different NT systems and emesis would contribute to the identification of personalized risk factors as well as help selecting the most efficient individualized antiemetic treatment for each patient, further enhancing the benefit and cost-effectiveness of genotyping.

## Data Availability

The datasets generated and analyzed during the current study are available in the Yareta repository, DOI: 10.26037/yareta:33pqmyb4kreulpvugeyh4q56vm.
