# Supplementary material for: Genetic Susceptibility Toward Nausea and Vomiting in Surgical Patients
Source: Front Genet. 2022 Jan 31;12:816908. doi: 10.3389/fgene.2021.816908 (PMC8842269; doi:10.3389/fgene.2021.816908)
Supplement: Supplementary file 1 [file DataSheet4.docx]

**Supplementary data S4 : Mixed model parameters for PONV occurrence**

Linear regression model using continuous parameters for age and opioid consumption

|  | OR | 95 % CI | P-value^a^ |
| --- | --- | --- | --- |
| Intercept | - | - | 0.023* |
| Gender (0 = male, 1 = female) | 4.19 | 2.90-6.11 | **5.20E-14***** |
| Age group (in years) | 0.98 | 0.97-0.98 | **0.002*** |
| Smoking (0 = yes, 1 = no) | 1.38 | 0.93-2.08 | 0.113 |
| Cannabis (0 = yes, 1 = no) | 1.15 | 0.52-2.58 | 0.737 |
| History of PONV (0 = no, 1 = yes) | 2.36 | 1.54-3.64 | **9.73E-05***** |
| Surgery (0 = other, 1 = visc, gyneco) | 0.73 | 0.50-1.05 | 0.090 |
| Volatile anesthetics (0 = no, 1 = yes) | 2.90 | 1.66-5.19 | **2.42E-04***** |
| Perioperative opioid consumption (in morphine eq.) | 0.99 | 0.99-1.00 | **0.003**** |

*^a^ Signif. codes: 0 ‘***’ 0.001 ‘**’ 0.01 ‘*’ 0.05*

The model characteristics using continuous numerical variables for age and opioid consumption has a residual deviance of 709.85 on 592 degrees of freedom for an AIC^1^ of 727.85, versus a residual deviance of 723.48 on 592 degrees of freedom and an AIC of 741.48 when all variables are dichotomous. Both models have a null deviance of 824.27 on 600 degrees of freedom
