# Supplementary material for: Genetic Susceptibility Toward Nausea and Vomiting in Surgical Patients
Source: Front Genet. 2022 Jan 31;12:816908. doi: 10.3389/fgene.2021.816908 (PMC8842269; doi:10.3389/fgene.2021.816908)
Supplement: Supplementary file 2 [file DataSheet7.docx]

**Supplementary data S7: Influence of dexamethasone treatment on logistic regression parameters for single SNPs and CYP activities**

Comparison for single nucleotide polymorphisms

| Gene | SNP ID | w/o dexa | | Dexa (y/n) | | | | Dexa category | | | |
| --- | --- | --- | --- | --- | --- | --- | --- | --- | --- | --- | --- |
|  |  | OR | P-value^4^ | OR | | P-value^4^ | | OR | P-value^4^ | | |
| *COMT* | *rs4680* | 0.92 | 0.693 | 0.92 | | | 0.692 | 0.92 | | 0.698 | |
|  | *rs4633* | 0.93 | 0.745 | 0.93 | | | 0.745 | 0.93 | | 0.750 | |
|  | *rs165722* | 0.95 | 0.800 | 0.95 | | | 0.800 | 0.95 | | 0.806 | |
|  | *rs6269* | 0.95 | 0.801 | 0.95 | | 0.801 | | 0.95 | 0.807 | | |
|  | *rs4818* | 0.89 | 0.594 | 0.89 | | 0.594 | | 0.89 | 0.601 | | |
| *CHRM3* | *rs2165870* | 0.97 | 0.878 | 0.97 | | 0.875 | | 0.97 | 0.884 | | |
|  | *rs10802789* | 1.05 | 0.797 | 1.05 | | 0.796 | | 1.06 | 0.790 | | |
|  | *rs685550* | 1.25 | 0.371 | 1.25 | | 0.371 | | 1.25 | 0.368 | | |
| *HTR1A* | *rs6295* | 0.88 | 0.550 | 0.88 | | 0.537 | | 0.88 | 0.537 | | |
| *HTR2A* | *rs6313* | **0.62** | **0.022** | **0.61** | | **0.022** | | **0.62** | **0.023** | | |
| *HTR3A* | *rs10160548* | 1.24 | 0.321 | 1.24 | | 0.321 | | 1.23 | 0.329 | | |
|  | *rs1985242* | 0.94 | 0.772 | 0.94 | | 0.768 | | 0.93 | 0.750 | | |
|  | *rs1176713* | 0.93 | 0.782 | 0.94 | | 0.785 | | 0.94 | 0.783 | | |
| *HTR3B* | *rs1176744* | 1.10 | 0.700 | 1.10 | | 0.702 | | 1.11 | 0.677 | | |
|  | *rs3758987* | 1.05 | 0.838 | 1.05 | | 0.839 | | 1.06 | 0.824 | | |
|  | *rs1672717* | **0.63** | **0.043** | **0.62** | | **0.040** | | **0.62** | **0.040** | | |
|  | *rs3782025* | **0.66** | **0.052** | **0.66** | | **0.049** | | **0.66** | **0.049** | | |
|  | *rs76124337* | 0.67 | 0.079 | 0.67 | | 0.078 | | 0.67 | 0.078 | | |
|  | *rs45460698* | 0.92 | 0.772 | 0.92 | | 0.773 | | 0.92 | 0.777 | | |
| *HTR3D* | *rs6443930* | 1.26 | 0.275 | 1.26 | 0.275 | | | 1.26 | | | 0.280 |
| *OPRM1* | *rs1799971* | 0.98 | 0.949 | 0.98 | 0.945 | | | 0.98 | | | 0.941 |
| *DRD2* | *rs1800497* | 1.31 | 0.350 | 1.31 | 0.351 | | | 1.30 | | | 0.375 |
| *TACR1* | *rs3755468* | 1.01 | 0.978 | 1.01 | 0.976 | | | 1.01 | | | 0.981 |
| *FAAH* | *rs324420* | 0.63 | 0.076 | 0.63 | 0.076 | | | 0.64 | | | 0.078 |
| *ABCB1* | *rs1128503* | 0.91 | 0.679 | 0.95 | 0.816 | | | 0.92 | | | 0.707 |
|  | *rs1045642* | 0.89 | 0.578 | 0.89 | 0.581 | | | 0.90 | | | 0.616 |
|  | *rs2032582^2^* | 1.06 | 0.782 | 1.06 | 0.782 | | | 1.07 | | | 0.756 |

Comparison for cytochrome P450 activity groups

| Gene | Determinant | w/o dexa | | Dexa (y/n) | | | Dexa category | | |
| --- | --- | --- | --- | --- | --- | --- | --- | --- | --- |
|  |  | OR | P-value^4^ | OR | P-value^4^ | | OR | P-value^4^ | |
| *CYP2D6* | Activity score | 0.88 | 0.594 | 0.88 | | 0.575 | 0.87 | | 0.548 |
| *CYP3A* | Activity group | 0.80 | 0.418 | 0.80 | | 0.418 | 0.80 | | 0.417 |
| *CYP2C9* | Nbr. of reduced allele | 1.42 | 0.249 | 1.42 | | 0.248 | 1.41 | | 0.256 |
| *CYP2C19* | Activity score | 1.00 | 0.987 | 1.00 | 0.980 | | 0.98 | 0.908 | |
| *CYP1A2* | Activity score | **0.55** | **0.012** | **0.54** | **0.012** | | **0.55** | **0.013** | |
| *CYP2B6* | Activity score | 1.12 | 0.578 | 1.12 | 0.581 | | 1.11 | 0.603 | |
