# Supplementary material for: Genetic Susceptibility Toward Nausea and Vomiting in Surgical Patients
Source: Front Genet. 2022 Jan 31;12:816908. doi: 10.3389/fgene.2021.816908 (PMC8842269; doi:10.3389/fgene.2021.816908)
Supplement: Supplementary file 3 [file DataSheet6.docx]

**Supplementary data S6: Logistic regression model including dexamethasone treatment in prediction of PONV recurrence**

A. Description population receiving study treatment:

The PONV recurrence cohort is composed of 229 individuals with PONV symptoms who were randomized to receive study treatment and who were subsequently followed for 24 hours. From the 264 patients suffering from PONV in the initial cohort, 33 did not receive study treatment and the recurrence of PONV was not documented for 2 who received it.

Table A: Population characteristics

|  | All | Dexamethasone group | | | |
| --- | --- | --- | --- | --- | --- |
| Drug dosage |  | 0 mg | 3 mg | 6 mg | 12 mg |
| Total population (Nbr.) | 229 | 56 | 53 | 61 | 59 |
| Female (%) | 73.8 | 73.2 | 71.7 | 73.8 | 76.3 |
| < 50 years (%) | 62.0 | 51.8 | 69.8 | 63.9 | 62.7 |
| Nonsmoking (%) | 72.1 | 82.1 | 73.6 | 60.7 | 72.9 |
| No cannabis (%) | 95.2 | 89.3 | 100.0 | 96.7 | 94.9 |
| With PONV history (%) | 34.9 | 42.9 | 34.0 | 39.3 | 23.7 |
| Visc. or gyneco. surgery (%) | 38.0 | 44.6 | 41.5 | 34.4 | 32.2 |
| With volatile anesthesia (%) | 91.7 | 85.7 | 94.3 | 91.8 | 94.9 |
| With high opioid (%)^a^ | 48.0 | 57.1 | 43.4 | 47.5 | 44.1 |

*^a^ Fisher exact test indicates that the difference in high opioid consumption between the groups is not statistically significant (p-value = 0.486).*

B. Parameter modelling including dexamethasone treatment (logistic regression)

Two different data modelling techniques were compared:

1. Patients having received dexamethasone versus those that did not receive the drug (0 = no, 1 = yes)

ie. there is a dichotomization of the cohort with 56 patients without dexamethasone versus 173 patients having received either one of the treatment doses of dexamethasone (3,6 or 12 mg).

1. Dexamethasone treatment group is considered as a categorical variable (without = 0; with 3mg = 1, with 6 mg = 2, with 12 mg = 3).

Table B: PONV association parameters

|  | Dexa (yes/no) | | | Dexa groups (0, 3, 6 or 12 mg) | | |
| --- | --- | --- | --- | --- | --- | --- |
|  | OR | 95 % CI | P-value^a^ | OR | 95 % CI | P-value^a^ |
| Gender (0 = male, 1 = female) | 0.92 | 0.46-1.79 | 0.802 | 0.93 | 0.46-1.81 | 0.823 |
| Age group (0 ≥ 50, 1 < 50 years) | 1.36 | 0.73-2.51 | 0.330 | 1.36 | 0.73-2.51 | 0.327 |
| Smoking (0 = yes, 1 = no) | 1.55 | 0.78-3.04 | 0.207 | 1.52 | 0.77-2.97 | 0.223 |
| Cannabis (0 = yes, 1 = no) | 1.29 | 0.28-5.24 | 0.726 | 1.32 | 0.30-5.22 | 0.696 |
| History of PONV (0 = no, 1 = yes) | 1.85 | 0.97-3.67 | 0.069 | 1.81 | 0.94-3.60 | 0.087 |
| Surgery (0 = other, 1 = visc, gyneco) | 0.70 | 0.38-1.28 | 0.244 | 0.69 | 0.37-1.26 | 0.225 |
| Volatile anesthetics (0 = no, 1 = yes) | 0.93 | 0.30-2.65 | 0.892 | 0.94 | 0.30-2.68 | 0.908 |
| High opioid (0 = no, 1 = yes) | 0.35 | 0.19-0.64 | **7.26E-04***** | 0.35 | 0.19-0.63 | **6.30E-04**** |
| Dexamethasone treatment | 0.97 | 0.47-1.96 | 0.929 | 0.93 | 0.71-1.22 | 0.614 |

*Signif. codes: 0 ‘***’ 0.001 ‘**’ 0.01 ‘*’ 0.05*
