# Supplementary material for: Genetic Susceptibility Toward Nausea and Vomiting in Surgical Patients
Source: Front Genet. 2022 Jan 31;12:816908. doi: 10.3389/fgene.2021.816908 (PMC8842269; doi:10.3389/fgene.2021.816908)
Supplement: Supplementary file 4 [file DataSheet3.DOCX]

**Supplementary data S3 : CYP450 metabolizer profile of the DexPONV study population**

Comparison of predicted CYP activity between the study and the general EUR populations

| % | *CYP2D6* | | *CYP2C9* | | *CYP2C19* | | *CYP3A* | |
| --- | --- | --- | --- | --- | --- | --- | --- | --- |
|  | **DexPONV** | **EUR^1^** | **DexPONV** | **EUR^2^** | **DexPONV** | **EUR^3^** | **DexPONV** | **EUR^4^** |
| UM | 5.0 | 5-10 | - | - | 30.3 | 20 | - | - |
| EM | 80.5 | 65-80 | 66.1 | 64.1 | 67.7 | 77 | 22.3 | 15.6 |
| IM | 8.7 | 10-15 | 30.7 | 33.3 |  |  | 68.3 | 75.6 |
| PM | 5.7 | 5-10 | 3.2 | 2.7 | 2.0 | 3 | 9.4 | 8.8 |

^1^ (1), ^2^ (2), ^3^ (3), ^4^ (4), note EUR distribution calculated on 262 patients.

| SNP ID |  | Allele | MAF *CYP1A2* SNPs^5^ | |
| --- | --- | --- | --- | --- |
|  |  |  | **DexPONV** | **MAF** |
| *rs762551* | *1F | C | 0.32 | 0.32 |
| *rs2069514* | *1C | A | 0.04 | 0.02 |

| SNP ID |  | Allele | MAF *CYP2B6* SNPs^5^ | |
| --- | --- | --- | --- | --- |
|  |  |  | **DexPONV** | **MAF** |
| *rs3745274* | *6 | T | 0.26 | 0.24 |
| *rs34223104* | *22 | C | 0.02 | 0.01 |
| *rs3211371* | *5 | T | 0.10 | 0.11 |
| *rs2279343* | *4 | G | 0.28 | 0.25^6^ |

^5^ (5), ^6^ (6)

References:

1. Gaedigk A, Simon SD, Pearce RE, Bradford LD, Kennedy MJ, Leeder JS. The CYP2D6 activity score: translating genotype information into a qualitative measure of phenotype. Clin Pharmacol Ther (2008) 83(2):234-42.

2. Budd WT, Meyers G, Dilts JR, O'Hanlon K, Woody JR, Bostwick DG, et al. Next generation sequencing reveals disparate population frequencies among cytochrome P450 genes: clinical pharmacogenomics of the CYP2 family. Int J Computational Biology and Drug Design (2016) 9(1/2):54-86.

3. Fricke-Galindo I, Cespedes-Garro C, Rodrigues-Soares F, Naranjo ME, Delgado A, de Andres F, et al. Interethnic variation of CYP2C19 alleles, 'predicted' phenotypes and 'measured' metabolic phenotypes across world populations. Pharmacogenomics J (2016) 16(2):113-23.

4. Lloberas N, Elens L, Llaudo I, Padulles A, van Gelder T, Hesselink DA, et al. The combination of CYP3A4*22 and CYP3A5*3 single-nucleotide polymorphisms determines tacrolimus dose requirement after kidney transplantation. Pharmacogenet Genomics (2017) 27(9):313-22.

5. Howe KL, Achuthan P, Allen J, Allen J, Alvarez-Jarreta J, Amode MR, et al. Ensembl 2021. Nucleic Acids Res (2021) 49(D1):D884-D91.

6. Karczewski KJ, Francioli LC, Tiao G, Cummings BB, Alfoldi J, Wang Q, et al. The mutational constraint spectrum quantified from variation in 141,456 humans. Nature (2020) 581(7809):434-43.
