# Supplementary figures and images for: Genetic Susceptibility Toward Nausea and Vomiting in Surgical Patients
Source: Front Genet. 2022 Jan 31;12:816908. doi: 10.3389/fgene.2021.816908 (PMC8842269; doi:10.3389/fgene.2021.816908)

**Supplementary data S9 –*HTR3B* polymorphisms versus age**


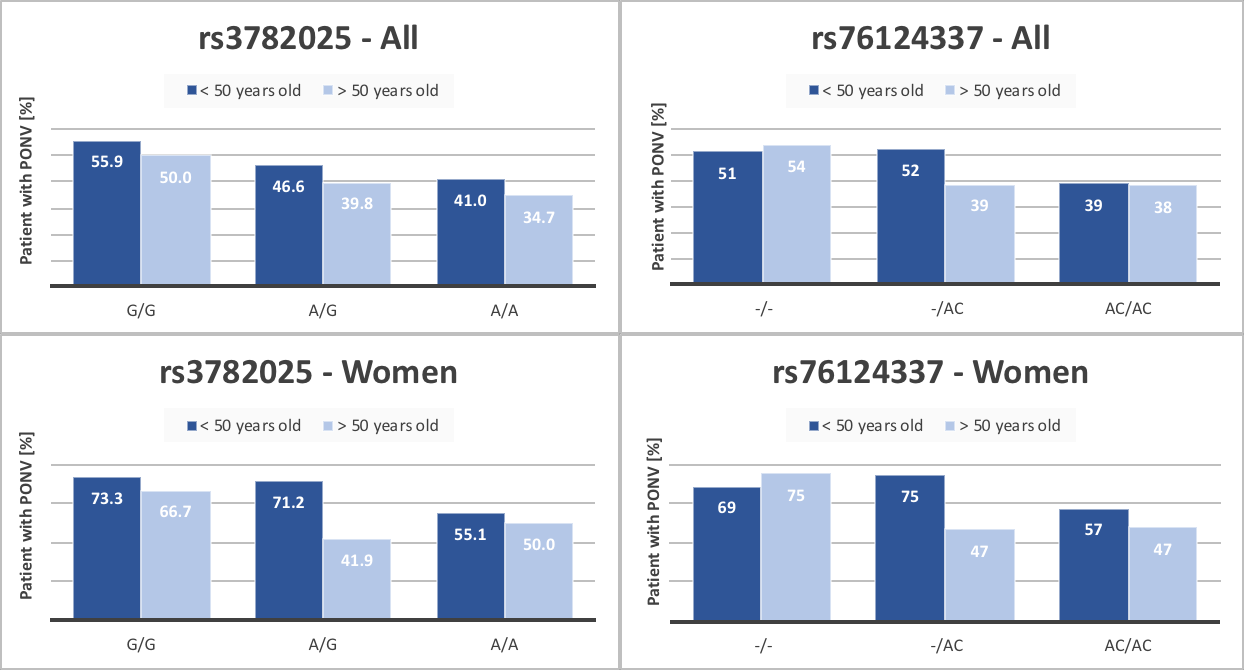

Supplement: Supplementary file 5 [file DataSheet9.DOCX]
