# Supplementary material for: Genetic Susceptibility Toward Nausea and Vomiting in Surgical Patients
Source: Front Genet. 2022 Jan 31;12:816908. doi: 10.3389/fgene.2021.816908 (PMC8842269; doi:10.3389/fgene.2021.816908)
Supplement: Supplementary file 6 [file DataSheet5.docx]

**Supplementary data S5 : Co-variable inter-dependence**

Table: p-value for Fisher exact test and logistic regression model vif^a^ scores

|  | Age | Gender | Smoking | Cannabis | History | Surgery | Vol. anesth. | High opioid | vif^a^ occurrence | vif^a^ recurrence |
| --- | --- | --- | --- | --- | --- | --- | --- | --- | --- | --- |
| Age | na | 2.70E-01 | **1.42E-03** | **1.25E-04** | 4.23E-02 | 7.11E-03 | 6.10E-02 | 1.000 | 1.073 | 1.027 |
| Gender |  | na | 4.31E-01 | 1.09E-02 | **2.90E-07** | 5.07E-01 | **3.10E-04** | 1.20E-01 | 1.081 | 1.048 |
| Smoking |  |  | na | **9.60E-10** | 7.59E-01 | 4.77E-01 | 6.92E-01 | 8.09E-02 | 1.088 | 1.110 |
| Cannabis |  |  |  | na | 1.00E+00 | 1.72E-01 | 6.10E-01 | 1.34E-01 | 1.102 | 1.082 |
| History of PONV |  |  |  |  | na | 7.94E-02 | 2.13E-03 | 8.48E-01 | 1.066 | 1.019 |
| Surgery |  |  |  |  |  | na | 1.70E-02 | 2.13E-03 | 1.046 | 1.031 |
| Vol. anesth. | - | - | - | - | - | - | na | 3.58E-02 | 1.096 | 1.088 |
| High opioid | - | - | - | - | - | - | - | na | 1.038 | 1.055 |

Bonferroni correction for multiple analysis = 0.05 / 27 = 2E-03 ie all pair with a p-value below the limit (highlighted in the table) are significantly correlated to each other.

^a^ The vif (Variance Inflation Factor) is a measure of the severity of the collinearity between multiple factors of a linear regression, where co-variable independence is a starting requirement. A vif < 4 is generally considered acceptable.
