# Supplementary material for: Genetic Susceptibility Toward Nausea and Vomiting in Surgical Patients
Source: Front Genet. 2022 Jan 31;12:816908. doi: 10.3389/fgene.2021.816908 (PMC8842269; doi:10.3389/fgene.2021.816908)
Supplement: Supplementary file 7 [file DataSheet10.DOCX]

**Supplementary data S10: Genotyping model for *HTR3B* polymorphisms**

| SNP ID | Major allele | Minor allele | Model | OR | 95 % CI' | P-value^1^ |
| --- | --- | --- | --- | --- | --- | --- |
| *rs3758987* | T | C | **DOM** | 0.63 | 0.44-0.90 | **0.011*** |
|  |  |  | REC | 0.89 | 0.45-1.77 | 0.741 |
|  |  |  | ADD | 0.73 | 0.55-0.97 | **0.033*** |
| *rs45460698* | AAG | - | DOM | 0.80 | 0.53-1.21 | 0.289 |
|  |  |  | REC | 1.04 | 0.29-3.71 | 0.955 |
|  |  |  | ADD | 0.84 | 0.58-1.21 | 0.352 |
| *rs1176744* | A (TYR) | C (SER) | **DOM** | 0.67 | 0.47-0.96 | **0.028*** |
|  |  |  | REC | 0.82 | 0.44-1.50 | 0.516 |
|  |  |  | ADD | 0.76 | 0.58-1.00 | 0.051 |
| *rs76124337* | CA | - | **DOM** | 1.63 | 1.14-2.34 | **0.008**** |
|  |  |  | REC | 1.66 | 0.95-2.90 | 0.074 |
|  |  |  | **ADD** | 1.47 | 1.12-1.91 | **0.005**** |
| *rs3782025* | A | G | DOM | 1.51 | 1.03-2.23 | **0.034*** |
|  |  |  | REC | 1.65 | 1.06-2.57 | **0.026*** |
|  |  |  | **ADD** | 1.40 | 1.09-1.79 | **0.009**** |
| *rs1672717* | A | G | **DOM** | 1.70 | 1.18-2.44 | **0.004**** |
|  |  |  | REC | 1.47 | 0.87-2.50 | 0.150 |
|  |  |  | **ADD** | 1.45 | 1.12-1.89 | **0.005**** |

^1^ Signif. codes: 0 ‘***’ 0.001 ‘**’ 0.01 ‘*’ 0.05
