# Supplementary material for: Genetic Susceptibility Toward Nausea and Vomiting in Surgical Patients
Source: Front Genet. 2022 Jan 31;12:816908. doi: 10.3389/fgene.2021.816908 (PMC8842269; doi:10.3389/fgene.2021.816908)
Supplement: Supplementary file 8 [file DataSheet2.DOCX]

**Supplementary data S2 : Genomic integrity of the study cohort**

Figure A: Minor allele frequencies (MAF) of study vs general population


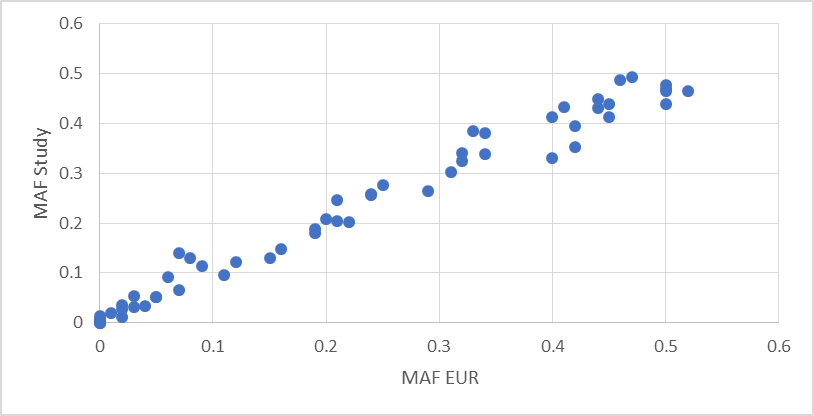


Figure B: Minor allele frequencies (MAF) of case vs controls within study.


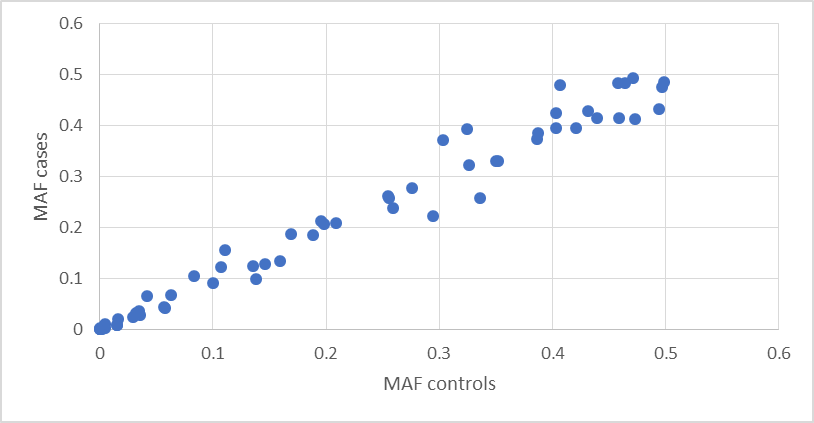


The minor allele frequencies (MAFs) observed in the study cohort are consistent with those of a standard population of European descent (according to the Ensembl.org repository [1]) (Figure A) and there are no major deviations between the MAF of cases versus controls (Figure B). Note that we used gnomADg (genomes r3.0) [2] as reference for *rs2279343* instead of the Ensmbl.org repository based on the gnomAD exon.

References:

1. Howe, K.L., et al., Ensembl 2021. Nucleic Acids Res, 2021. 49(D1): p. D884-D891.

2. Karczewski, K.J., et al., The mutational constraint spectrum quantified from variation in 141,456 humans. Nature, 2020. 581(7809): p. 434-443.
