# Supplementary material for: Genetic Susceptibility Toward Nausea and Vomiting in Surgical Patients
Source: Front Genet. 2022 Jan 31;12:816908. doi: 10.3389/fgene.2021.816908 (PMC8842269; doi:10.3389/fgene.2021.816908)
Supplement: Supplementary file 9 [file DataSheet8.DOCX]

**Supplementary data S8 – Confounding analysis**

For each polymorphism, we tested the effect of the genetic data on the parameters of all other statistically significant (ie *p*> 0.05) co-variables to detect possible interferences and possibly unravel some mode of action of the SNP. The confounder analysis was considered significant if the change in the estimation of the regression parameter >13%. Only three events were identified and the corresponding regressions are displayed in Table A. The vif scores of each of the parameters of the concerned equations were all below 1.2, indicating that the mathematical integrity of those models remains valid (Table B).

The impact of the two of the *HTR3B* polymorphism on the age association with PONV occurrence is discussed in the result section. The relationship between *TARC1* and surgery might result from a distribution bias. Indeed, our initial testing hypothesis includes that patients with different genotypes are evenly distributed among the different categories. However, as patients in the cohort were not randomized according to their genetic trait and the proportion of patients from the whole cohort with *TACR1* *rs3755468* CT genotype was considerably lower in the group undergoing visceral or gynecological surgeries (33%).

Table A: Logistic regression model parameters for PONV occurrence with significant confounding effect

|  | Starting values | | *rs3782025* | | | *rs76124337* | | | *rs3755468* | | |
| --- | --- | --- | --- | --- | --- | --- | --- | --- | --- | --- | --- |
|  | OR | P-value | OR | P-value | % Conf | OR | P-value | % Conf | OR | P-value | % Conf |
| SNP | - |  | 1.40 | 0.009 |  | 1.47 | 0.005 |  | 0.77 | 0.032 |  |
| Gender (0 = male, 1 = female) | 4.11 | 6.02E-14 | 4.01 | 3.12E-13 | -1.7 | 4.26 | 3.39E-14 | 2.6 | 4.40 | 2.58E-14 | 4.8 |
| Age group (0 ≥ 50, 1 < 50 years) | 1.52 | 0.026* | **1.64** | **0.010** | **18.5** | **1.61** | **0.013** | **13.8** | 1.53 | 0.026 | 2.3 |
| Smoking (0 = yes, 1 = no) | 1.35 | 0.140 | 1.40 | 0.099 | nd | 1.36 | 0.132 | nd | 1.33 | 0.165 | nd |
| Cannabis (0 = yes, 1 = no) | 1.08 | 0.849 | 1.09 | 0.823 | nd | 1.11 | 0.790 | nd | 0.98 | 0.953 | nd |
| History of PONV (0 = no, 1 = yes) | 2.38 | 6.87E-05 | 2.54 | 2.57E-05 | 7.7 | 2.35 | 1.06E-04 | -1.6 | 2.30 | 1.73E-04 | -3.8 |
| Surgery (0 = other, 1 = visc, gyneco) | 0.72 | 0.082 | 0.74 | 0.109 | nd | 0.71 | 0.076 | nd | **0.68** | **0.045** | **16.9** |
| Volatile anesthetics (0 = no, 1 = yes) | 2.88 | 2.30E-04 | 2.82 | 3.43E-04 | -1.9 | 2.80 | 3.59E-04 | -2.6 | 2.71 | 7.29E-04 | -5.6 |
| High opioid (0 = no, 1 = yes) | 0.85 | 0.378 | 0.86 | 0.432 | nd | 0.85 | 0.368 | nd | 0.83 | 0.320 | nd |

nd = not determined (p-value > 0.05)

Table B: Vif score for the parameters of the equations with significant genetic confounding effect

|  | *rs3782025* occurrence | *rs76124337* occurrence | *rs3755468* occurrence |
| --- | --- | --- | --- |
| Age | 1.093 | 1.079 | 1.072 |
| Gender | 1.079 | 1.097 | 1.105 |
| Smoking | 1.093 | 1.088 | 1.098 |
| Cannabis | 1.106 | 1.104 | 1.119 |
| History | 1.071 | 1.066 | 1.062 |
| Surgery | 1.054 | 1.053 | 1.055 |
| Vol. anesth. | 1.100 | 1.095 | 1.092 |
| High opioid | 1.045 | 1.041 | 1.044 |
| Genetic factor | 1.025 | 1.021 | 1.037 |

^1^ The vif (Variance Inflation Factor) is a measure of the severity of the collinearity between multiple factors of a linear regression, where co-variable independence is a starting requirement. A vif < 4 is generally considered acceptable.
