# Supplementary material for: Genetic Susceptibility Toward Nausea and Vomiting in Surgical Patients
Source: Front Genet. 2022 Jan 31;12:816908. doi: 10.3389/fgene.2021.816908 (PMC8842269; doi:10.3389/fgene.2021.816908)
Supplement: Supplementary file 10 [file DataSheet12.DOCX]

**Appendix 12: ROC curve analysis**

ROC analyses were performed using the EasyROC tool [1], available at : http://www.biosoft.hacettepe.edu.tr/easyROC/.

1. ROC curves for full cohort:

| Marker | AUC | SE | | Lower CI 95% | Upper CI 95% | z | | P-value | |
| --- | --- | --- | --- | --- | --- | --- | --- | --- | --- |
| Apfel (“classic score”) | 0.633 | 0.022 | 0.591 | | 0.676 | | 6.122 | 9.23E-10 |  |
| Model 1 | 0.660 | 0.022 | 0.617 | | 0.702 | | 7.385 | 1.52E-13 |  |
| Model 2 | 0.665 | 0.022 | 0.623 | | 0.708 | | 7.665 | 1.78E-14 |  |

1. ROC curves for category 2 women

| Marker | AUC | SE | | Lower CI 95% | Upper CI 95% | z | | P-value | |
| --- | --- | --- | --- | --- | --- | --- | --- | --- | --- |
| Apfel (“classic score”) | 0.500 | 0.000 | 0.500 | | 0.500 | | - | - |  |
| Model 1 | 0.645 | 0.042 | 0.562 | | 0.728 | | 3.438 | 5.87E-04 |  |
| Model 2 | 0.668 | 0.043 | 0.584 | | 0.753 | | 3.900 | 9.62E-05 |  |

1. ROC curves for category 2 women < 50 years old

| Marker | AUC | SE | Lower CI 95% | Upper CI 95% | z | | P-value | |
| --- | --- | --- | --- | --- | --- | --- | --- | --- |
| Apfel (“classic score”) | 0.500 | 0.000 | 0.500 | 0.500 | | - | - |  |
| Model 1 | 0.648 | 0.056 | 0.538 | 0.759 | | 2.641 | 8.27E-03 |  |

Reference:

[1] Goksuluk, D., et al., *easyROC: An Interactive Web-tool for ROC Curve Analysis Using R Language Environment.* The R Journal, 2016. **8**(2): p. 213-230.
