# Supplementary material for: Genetic Susceptibility Toward Nausea and Vomiting in Surgical Patients
Source: Front Genet. 2022 Jan 31;12:816908. doi: 10.3389/fgene.2021.816908 (PMC8842269; doi:10.3389/fgene.2021.816908)
Supplement: Supplementary file 11 [file DataSheet11.DOCX]

**Supplementary data S11: Comparison of logistic regression parameters for *HTR3B* mutations in patient receiving or not tramadol**

| Gene | SNP ID | with tramadol | | without tramadol | | All | |
| --- | --- | --- | --- | --- | --- | --- | --- |
|  |  | OR | P-value^1^ | OR | P-value^1^ | OR | P-value^1^ |
| *HTR3B* | *rs1176744* | 0.43 | 0.253 | 0.82 | 0.187 | 0.76 | 0.051 |
|  | *rs3758987* | 0.28 | 0.132 | 0.80 | 0.148 | 0.73 | **0.033*** |
|  | *rs1672717* | 0.67 | 0.520 | 1.51 | **0.004**** | 1.45 | **0.005**** |
|  | *rs3782025* | 0.34 | 0.206 | 1.46 | **0.005**** | 1.40 | **0.009**** |
|  | *rs76124337* | 0.58 | 0.404 | 1.54 | **0.003**** | 1.47 | **0.005**** |
|  | *rs45460698* | 2.86 | 0.459 | 0.76 | 0.154 | 0.84 | 0.352 |

^1^ Signif. codes: 0 ‘***’ 0.001 ‘**’ 0.01 ‘*’ 0.05
