# Supplementary material for: Genetic Susceptibility Toward Nausea and Vomiting in Surgical Patients
Source: Front Genet. 2022 Jan 31;12:816908. doi: 10.3389/fgene.2021.816908 (PMC8842269; doi:10.3389/fgene.2021.816908)
Supplement: Supplementary file 12 [file DataSheet1.docx]

**Supplementary material S1: Single nucleotides polymorphisms for CYP activity determination**

| Gene | SNP ID | Chr | Major allele | Minor allele | Corresponding Star allele^1^ | MAF EUR^2^ | MAF Study |
| --- | --- | --- | --- | --- | --- | --- | --- |
| *CYP2D6* | *rs5030656* | 22 | TCT | - | *9 | 0.03 | 0.03 |
|  | *rs769258* | 22 | C | T | *35 | 0.05 | 0.04 |
|  | *rs1065852* | 22 | G | A | *4, *10 | 0.20 | 0.21 |
|  | *rs28371706* | 22 | G | A | *17 | 0.00 | 0.01 |
|  | *rs5030655* | 22 | A | - | *6 | 0.02 | 0.02 |
|  | *rs5030865* | 22 | C | A/T | *8 | 0.00/0.00 | 0.00/0.00 |
|  | *rs3892097* | 22 | C | T | *4 | 0.19 | 0.19 |
|  | *rs35742686* | 22 | T | - | *3 | 0.02 | 0.01 |
|  | *rs16947* | 22 | G | A | *2, *4, *8, *17, *29, *35, *41 | 0.34 | 0.37 |
|  | *rs5030867* | 22 | T | G | *7 | 0.00 | 0.00 |
|  | *rs28371725* | 22 | C | T | *41 | 0.09 | 0.12 |
|  | *rs59421388* | 22 | C | T | *29 | 0.00 | 0.01 |
|  | *rs1135840* | 22 | G | C | *2, *4, *6, *8, *10, *17, *29, *35, *41 | 0.45 | 0.42 |
| *CYP2C19* | *rs4244285* | 10 | G | A | *2 | 0.15 | 0.12 |
|  | *rs4986893* | 10 | G (TRP) | A (*) | *3 | 0.00 | 0.00 |
|  | *rs12248560* | 10 | C | T | *17 | 0.22 | 0.21 |
| *CYP1A2* | *rs2069514* | 15 | G | A | *1C | 0.02 | 0.04 |
|  | *rs762551* | 15 | A | C | *1F | 0.32 | 0.32 |
| *CYP3A4* | *rs35599367* | 7 | G | A | *22 | 0.05 | 0.04 |
|  | *rs2740574* | 7 | T | C | *1B | 0.03 | 0.07 |
|  | *rs2242480* | 7 | C | T | *1G | 0.08 | 0.16 |
| *CYP3A5* | *rs776746* | 7 | C | T | *3 | 0.06 | 0.10 |
|  | *rs10264272* | 7 | C | T | *6 | 0.00 | 0.00 |
| *CYP3A7* | *rs45446698* | 7 | T | G | *1C | 0.04 | 0.03 |
| *CYP2C9* | *rs1799853* | 10 | C (ARG) | T (CYS) | *2 | 0.12 | 0.10 |
|  | *rs1057910* | 10 | A (ILE) | C (LEU) | *3 | 0.07 | 0.07 |
| *CYP2B6* | *rs3745274* | 19 | G | T | *6, *7, *9, *36 | 0.24 | 0.26 |
|  | *rs34223104* | 19 | T | C | *22, *36 | 0.01 | 0.02 |
|  | *rs3211371* | 19 | C | T | *5, *7 | 0.11 | 0.09 |
|  | *rs2279343* | 19 | A | G | *4, *6, *7, *36 | 0.25^3^ | 0.28 |

^1^ Note: The same polymorphism can belong to more than one star allele.

^2^ Howe, K.L., et al., Ensembl 2021. Nucleic Acids Res, 2021. 49(D1): p. D884-D891.

^3^ Karczewski, K.J., et al., The mutational constraint spectrum quantified from variation in 141,456 humans. Nature, 2020. 581(7809): p. 434-443.
